# Incidence and economic burden of community-acquired gastroenteritis in the Netherlands: Does having children in the household make a difference?

**DOI:** 10.1371/journal.pone.0217347

**Published:** 2019-05-23

**Authors:** Roan Pijnacker, Marie-Josée J. Mangen, Gerrita van den Bunt, Eelco Franz, Wilfrid van Pelt, Lapo Mughini-Gras

**Affiliations:** 1 Center for Infectious Disease Control, National Institute for Public Health and the Environment (RIVM), Bilthoven, the Netherlands; 2 Julius Center for Health Sciences and Primary Care, University Medical Center Utrecht (UMCU), Utrecht, the Netherlands; 3 Institute for Risk Assessment Sciences, Utrecht University, Utrecht, the Netherlands; Uniformed Services University of the Health Sciences, UNITED STATES

## Abstract

This study aimed at estimating gastroenteritis (GE) incidence in all age groups of the Netherlands’ general population, with special emphasis on the role of children in GE burden, and the associated costs. Monthly from November 2014 to November 2016, a random sample of 2000 residents in the Netherlands was invited to complete a questionnaire on household characteristics and health complaints. We calculated GE incidence rates standardized to the Dutch population and used multivariable logistic regression models to identify potential risk factors. We calculated the costs related to resources used within the healthcare sector, the resources used by patients and their families, and productivity losses (paid worktime) due to GE. The overall standardized incidence rate was 0.81 GE episodes/person-year, with the highest rate in children ≤4 years (1.96 episodes/person-year). GE was observed more often in households with children (≤17 years), especially if children attended out-of-home childcare services, and among individuals with non-native Dutch ethnic background. Less GE was observed among employed persons aged 25–64 years, compared with those unemployed, but the opposite was observed in persons ≥65 years. The average costs per GE episode was €191, resulting in €945 million annual total costs for GE in the Netherlands (€55 per inhabitant). The majority of costs (55%) were attributable to productivity losses of the ill or their caregivers. In conclusion, GE still poses a significant burden, particularly in preschool children and adults living in households with children. Similar to other industrialized countries, the major factor driving the costs due to GE was the loss of productivity. This study also provides up-to-date baseline GE incidence rates and associated societal costs to better contextualize the burden of the disease in support of policy making.

## Introduction

Gastroenteritis (GE) ranks first in deaths caused by communicable diseases globally, although the numbers have reduced over the past decades [1]. In industrialized countries, however, the course of the disease is often mild and short-term [1–3]. Despite being rarely fatal, the morbidity and economic costs of GE are substantial due to its high incidence even in an industrialized country like the Netherlands [3–5]. Cost-of-illness (COI) studies are instrumental in public health policy and serve as important input for economic evaluations of possible intervention measures to support decision-making. They namely measure the costs related to resources used within the healthcare sector, the resources used by patients and their families, and productivity losses and other non-healthcare related resources used that are indirectly related to illness [6]. They highlight the magnitude of the impact of an illness on the society and allow for comparison of the relative burden of different (infectious) diseases.

In the Netherlands (~17 million population), the average costs of each GE episode was estimated at €77 in 2003, with total national costs of €345 million [4]. In 2009, the average costs per GE episode had increased to €133–€151, corresponding to national costs of €611–695 million [3]. This increase was mainly attributable to a rise in healthcare costs over the years. These two studies were based on GE incidence estimates from a prospective population-based cohort study conducted in 1999, reporting 283 GE episodes per 1,000 person-years [7]. The incidence was estimated to be more than 3 times higher in a retrospective population-based cohort study in 2009, with 964 GE episodes per 1,000 person-years [2]. This large difference was thought to be mainly due to the retrospective nature of the latter study, which was more sensitive to ‘telescoping’ bias, i.e. remembering a GE episode to be more recent than it actually was, and sample selection bias of those who recently experienced GE. It is likely that the average costs per GE episode are currently higher than was found in 2009 because healthcare costs have further increased [3, 6]. Since 2009, studies have focused on GE in young children and/or their parents in the Netherlands, and none estimated the GE incidence in all age groups [8, 9]. Moreover, previously published COI studies on GE in the Netherlands did not differentiate between households with and without children, whilst the incidence of GE is known to be highest in households with young children [9, 10], with parents and siblings of GE-affected children having an up to 4- and 8-fold increased risk for secondary GE, respectively [9, 11].

The overall aim of this study was to update and characterize estimates for community-acquired GE in all age groups of the general population in the Netherlands, with special emphasis on the role of children in GE burden in the household, including associated costs. This was achieved by (i) estimating GE incidences, (ii) identifying risk factors for GE, (iii) identifying differences in GE burden between households with and without children, and (iv) estimating the average economic costs of GE episodes.

## Materials and methods

### Study design

We used questionnaire data that was collected during a monthly-repeated cross-sectional survey on antimicrobial resistance that was performed among the general population in the Netherlands from November 2014 to November 2016 [12]. A random sample of 2000 Dutch residents, including all age groups, was drawn monthly from municipal population registries covering the whole national population with a maximum of one person per household. They were invited by regular mail to complete a web-based questionnaire. Invitees could fill in the questionnaire with the help of someone else, such as a family member or a healthcare worker. For children aged 0–12 years, a parent or caregiver was asked to fill out the questionnaire. Children aged 13–17 years could fill in the questionnaire themselves, but help from a parent was recommended.

### Questionnaire

The web-based questionnaire included questions about general demographics (e.g. age, gender, country of birth), education, employment, household characteristics (e.g. number of children, age of children in the household, presence of pet), out-of-home care of children (i.e. day-care attendance, kindergarten, guest parent), outdoor activities, health symptoms in the past four weeks (e.g. diarrhea, vomiting), chronic conditions (e.g. enteropathies, food allergy, immune disorder) the use of medication (e.g. antibiotics, antacid drugs, paracetamol), medical care (e.g. contact with the general practitioner (GP), hospitalization) and laboratory testing (S1 and S2 Questionnaires).

### Gastroenteritis case definition

A GE case was defined as a person with ≥3 diarrheal discharges or ≥1 episode of vomiting in 24 hours during the four weeks prior to completing the questionnaire, according to a commonly used GE case definition [13]. We excluded cases of probable non-infectious origin, i.e. those with underlying enteropathies. The same case definition was also used in previous studies estimating the incidence of GE in the general population in 2009 in the Netherlands, with the exception that we were not able to exclude cases with vomiting due to regurgitation, motion sickness/vertigo, nauseous event, traumatic event, or drug/alcohol abuse, as this information was not collected [2, 12].

### Statistical analysis

The representativeness of the sample of study participants was assessed in relation to the demographics of the Dutch general population, including age (five-year age groups; 0–4, 5–9, 10–14 years etc.), gender, and location of residence (urban: ≥2500 households/km^2^, intermediate: 500–2500 households/km^2^, rural: <500 households/km^2^). The cut-offs for location of residence were those adopted by the Dutch Central Bureau for Statistics (CBS) [14]. All data analyses were weighted to adjust for differences in the distributions by age, gender and location of residence between our study population and the general population the sample was drawn from.

The incidence of GE was expressed as the number of episodes per person-year (365/28 x 4-weekly GE incidence proportion), because the recall period was 28 days. GE incidence was reported by age groups (0–4, 5–17, 18–29, 30–44, 45–65, and 65+ years of age), gender, location of residence, and level of education. Age groups cut-offs were chosen according to previous cost-of-illness studies on GE in the Netherlands to ensure comparability [2, 3]. Age groups 18–29 (‘young adults’), 30–44 (‘adults’) and 45–65 (‘middle-aged’) years were stratified by the presence of children (defined as those aged 0–17 years) in the household to study differences between households with and without children. For all adult groups (defined as those aged ≥18 years) living in a household with children, no data was available on whether they were the parents of those children, or for example an older sibling or another relative. The level of education was categorized as low (primary, lower vocational or lower secondary education), intermediate (intermediate vocational, intermediate secondary or higher secondary education), and high (higher vocational and university education). For those younger than 18 years who did not fill in the questionnaire themselves, the education level of the person filling it in was used.

An overall multivariable weighted logistic regression model was built to assess the effect of age (0–4, 5–17, 18–29, 30–44, 45–65, and 65+ years of age), gender, location of residence (rural, intermediate and urban), and educational level (low, intermediate, high) on GE. In addition, a multivariable weighted logistic regression model was built for each age group to assess the effect of the number of children in the household (one, two, or ≥three children, with ‘no children’ as reference group), the age of children in the household (0, 1–2, 3–12, and 13–17 years) and their attendance of out-of-home care (i.e. day-care, kindergarten, guest parent, with “no out-of-home-care” as reference group) on GE. Location of residence, educational level, employment, gender, country of birth (born in- or outside the Netherlands), and comorbidities (food allergy, immune disorder, asthma, and cancer) were considered as potential confounders. They were kept in the model when a change of more than 10% in the coefficients of the other covariates was observed when removed from the model, regardless of significance. We also tested variables for biologically plausible interactions as reported previously [9]. Selection between collinear variables was based on improved model fit using the Akaike information criteria (AIC). A backward stepwise variable procedure was applied, where variables with a p-value <0.05 were kept in the model. Associations were expressed as odds ratios (OR) with corresponding 95% confidence intervals (CI). Analyses were performed using STATA version 15.1 (College Station, TX, USA).

### Cost of illness

Costs of GE were divided into direct healthcare costs, patient and/or family costs and productivity losses, as described below. The average costs per GE episode, the average costs per Dutch inhabitant (total costs of GE/17.2 million inhabitants), as well as the total costs of GE, were calculated for age groups 0–4, 5–17, 18–29, 30–44, 45–65, and 65+ years of age. For age groups 18–29, 30–44 and 45–65 years, costs were further stratified by households with and without children. Unit costs were expressed in 2017 Euros (€) (S1 Table). Because retrospective surveys on disease burden are prone to overestimation of the incidence, we adjusted our GE estimates when calculating costs, based on findings from a community study on GE in the United Kingdom.[2, 15–20]. They found a GE estimate of 55/100 person-years in a retrospective survey, while it was 19.4/100 person-years (2.8 times lower) in a prospective study in the same study population [21]. Hence, to remain conservative, we divided all our GE estimates by a factor of 2.8 when calculating the average costs per inhabitant (regardless of GE) and the total costs. However, we also calculated the upper boundary of costs without applying the correction factor. The rationale is that over-selection of persons with GE might be less pronounced in our study than the retrospective study in the United Kingdom, because participants in our study were invited to participate in a study on antibiotic resistance and not on GE. Hence, persons with GE might have been equally motivated to participate in our study compared with persons without GE. Moreover, prospective studies are also subject to biases, including selection bias, which can potentially lead to under- or overestimation of the incidence, which affects the correction factor as well [22]. We report GE estimates without correction in order to ensure comparability with previous studies on GE community incidence, which were mainly retrospective in nature [2, 15–20].

#### Direct healthcare costs

Direct healthcare costs included costs for GP consultation, hospitalization, ambulance transport to the hospital, medicines prescribed by a doctor, and laboratory testing. Because we had data on whether respondents contacted the GP, but not whether they actually visited the GP, we used data on overall all-cause GE consultation rates in GPs in 2016 to scale the GP consultation rate in our study. These data were collected by routine electronic health record extractions from Dutch GPs participating in the Nivel Primary Care Database (~7% national coverage) [23]. Since the questionnaire collected no data on ambulance utilization and length of hospitalization, we used data from a previous study in patients hospitalized for GE in the Netherlands [3, 24]. They reported that 1% of hospitalized children younger than 18 years of age and 50% of hospitalized adults were transported to hospital by ambulance. The mean length of hospitalization was 2.9 days in children, 9.9 days in adults 18–64 years, and 12.3 days in persons 65+ years. For the first GP visit, we used a weighted cost consisting of a standard consultation (i.e. 90% of patients visiting the GP and in 10% of the cases a house visit was required), and an additional telephone consultation (97% of all GP visits) based on previous research [25]. For subsequent visits, we assumed only the costs for the standard consultation. Medicine costs were derived from retail prices and prescription drug dispensing fees in the official price list published by Care Institute Netherlands (in Dutch: ‘Zorginstituut Nederland’) [26]. The duration of medicine use was based on the recommendations in the package leaflet.

#### Patient and/or family costs

Patient and/or family costs were costs paid by the patients themselves and/or his family, and included travel costs to the GP or hospital, as well as over-the-counter (OTC) medicines. Based on previous research, we assumed that 77% of persons younger than 15 years and 97% of those older than 15 years used a car or public transport (50/50) to go to the GP [3]. We used the Dutch average distance of 1.1 kilometers to the GP and 7.0 kilometers to the hospital, as well as a parking fee when traveling by car [6]. Similar to medicines prescribed by a doctor, we derived the costs of OTC medicines from the official retail price list of Care Institute Netherlands, but without pharmacist dispensing fees [26].

#### Productivity losses

Productivity losses were costs of time spent away from paid employment by the patients themselves and/or their caregivers as a result of a GE episode. Because our study collected no data on absenteeism from work, we based the number of paid work hours missed per GE episode on previous Dutch studies [3, 24]. In children younger than 5 years, two hours of paid work were missed per GE episode for caregiving by their parents or a relative [3]. In the age group 18–64 years, 4.5 work hours were missed for each GE episode by themselves or a caregiver [3]. In persons 65 years and older, 0.4 hours of paid work were missed by a caregiver [24]. For children aged 5–17 years, in absence of available literature, we assumed that the amount of paid employment missed was 50% of that missed in children younger than 5 years, i.e. 1 hour per GE episode, in line with assumptions from previous research [24]. The hourly wage of paid employment was obtained per five-year age group (i.e. 15–19 years, 20–24 years etc.) [6, 27, 28].

Costs were expressed in 2017 euros, and where necessary, updated to 2017 using Dutch consumer Price index as reported by the Dutch Central Bureau for Statistics [29].

### Ethics statement

This study received ethics approval from the Medical Research Ethics Committee of Utrecht University (WAG/om/14/012490). Written informed consent was obtained from all participants. All participants gave consent and in the case of children, parents gave consent.

## Results

In total, 9,512 of the 48,024 invited individuals (response: 19.8%) filled in the questionnaire. Questionnaires of 256 participants were discarded because they were incompletely filled in regarding the variables used here, leaving 9,256 participants. Compared to the general population, our sample overrepresented females (our study population: 52.6% *vs*. general population: 50.2%) and individuals from rural areas (31.7% *vs*. 16.3%), and underrepresented individuals from urban areas (6.2% *vs*. 22.7%) (S2 Table). Moreover, the mean age in our study population was higher than in the general population (42.7 years *vs*. 41.5 years). These differences were adjusted for by performing weighted analyses.

### Gastroenteritis incidence

A total of 544/9,256 persons (5.9%, standardized rate: 6.2%, 95%CI 5.6–6.9) experienced GE in the four weeks before completing the questionnaires. We excluded 97 persons (15.1%) with GE because they suffered from underlying enteropathies. Of the 544 persons with GE, 69 (12.7%) had diarrhea and vomiting, 257 (47.2%) had diarrhea without vomiting, and 218 (40.1%) had vomiting without diarrhea. The most frequently reported additional symptoms were abdominal cramps (43.6%), nausea (39.7%), fever (18.9%), mucus in stool (4.8%), and blood in stool (2.4%).

The overall standardized incidence was 0.81 GE episodes per person-year (95%CI: 0.72–0.90) (Table 1). The incidence was highest in children ≤4 years (1.96 episodes/person-year, 95%CI: 1.51–2.42), and lowest incidence in persons aged 45–65 years (0.60 episodes/person-year, 95%CI: 0.48–0.71) and ≥65 years (0.46 episodes/person-year, 95%CI: 0.32–0.60).

**Table 1:** Incidence of gastroenteritis per person-year by sociodemographic variables in the general population in the Netherlands, November 2014 to November 2016.

	N	GE	Crude %	St.^a^ %	95%CI	GE/yr	95% CI	aOR^b^	95% CI
**Age in years**									
0–4	522	86	16.5	15.1	11.9–19.0	1.96	1.51–2.42	2.6	1.8–3.8
5–17	1,439	98	6.8	6.3	4.9–7.7	0.82	0.64–1.00	0.9	0.6–1.3
18–24	555	56	10.1	11.3	7.5–15.2	1.47	0.98–1.98	1.8	1.1–3.0
With children^c^	143	14	9.8	13.2	4.7–21.7	1.72	0.61–2.83	n.i	
Without children^c^	411	42	10.2	10.8	6.5–15.1	1.41	0.85–1.97	n.i	
25–44	1,784	109	6.1	6.5	4.9–8.1	0.85	0.64–1.06	Ref	
With children^c^	1,018	65	6.4	7.0	4.7–9.2	0.91	0.61–1.20	n.i	
Without children^c^	758	44	5.8	6.0	3.6–8.5	0.78	0.47–1.11	n.i	
45–64	2,915	132	4.5	4.6	3.7–5.5	0.60	0.48–0.71	0.7	0.5–0.9
With children^c^	799	28	3.5	3.6	2.2–5.0	0.46	0.28–0.65	n.i	-
Without children^c^	2,097	103	4.9	5.0	3.9–6.1	0.65	0.50–0.80	n.i	-
65+	1,874	61	3.3	3.5	2.6–4.8	0.46	0.32–0.60	0.5	0.3–0.7
**Gender**									
Male	4,389	241	5.5	5.9	5.0–7.0	0.77	0.64–0.90	Ref	-
Female	4,867	303	6.2	6.5	5.7–7.5	0.85	0.73–0.96	1.1	0.9–1.4
**Location of residence**									
Rural	2,937	169	5.8	5.4	4.7–6.3	0.71	0.60–0.81	Ref	-
Intermediate	5,747	336	5.9	6.1	5.5–6.8	0.79	0.71–0.88	1.1	0.9–1.4
Urban	572	39	6.8	7.1	5.2–9.8	0.93	0.63–1.23	1.2	0.8–1.8
**Education level**									
Low	2,933	164	5.6	5.9	4.9–7.1	0.77	0.63–0.91	Ref	-
Intermediate	2,929	179	6.1	6.4	5.3–7.7	0.84	0.68–0.99	0.8	0.6–1.1
High	3,160	183	5.8	6.0	5.0–7.2	0.78	0.64–0.94	0.8	0.6–1.1
**Overall**	9,256	544	5.9	6.2	5.6–6.9	0.81	0.72–0.90	-	-

^a^Standardized by gender, age (five-year age groups) and location of residence (urban, intermediate, rural)

^b^Adjusted for age, gender, location of residence, and educational level

^c^Children aged 0–17 years living in the same household

GE: gastroenteritis, 95%CI: 95% confidence intervals, aOR: adjusted odds ratio, n.i.: not included in the multivariable model

### Risk factors for gastroenteritis

In children ≤4 years of age, living in a household with other children of <1 year was a risk factor for GE (OR 3.2, 95%CI: 1.2–8.7) compared with having no other children in the household, whereas living in an urban area was associated with a lower risk (OR 0.2, 95%CI 0.1–0.9) (Table 2). In children aged 0–17 years, having an immune disorder or asthma were risk factors for GE (OR 8.4, 95%CI: 1.5–47 and OR 8.1, 95%CI 2.3–29, respectively).

**Table 2:** Multivariable weighted logistic regression models of factors associated with gastroenteritis in children, stratified by age groups.

	0–4 years (n = 493)			5–17 years (n = 1,296)		
	N (%GE)^a^	OR^a^	95%CI	N (%GE)	OR^a^	95% CI
Other children in household						
None	146 (10)	Ref		171 (6)	Ref	
0y	63 (23)	**3.2**	**1.2–8.7**	25 (9)	0.8	0.2–3.4
1-2y	71 (16)	2.0	0.8–4.7	37 (13)	1.0	0.3–3.5
3-12y	214 (14)	1.3	0.7–2.4	655 (7)	0.8	0.4–1.6
13-18y	7 (4)	0.8	0.1–7.3	408 (4)	0.6	0.2–1.6
Out-of-house care^b^		-			1.7	0.8–3.7
Location of residence						
Rural	177 (18)	Ref		490 (7)	Ref	
Intermediate	303 (17)	1.0	0.6–1.6	882 (7)	0.9	0.6–1.5
Urban	42 (10)	**0.2**	**0.1–0.9**	67 (3)	0.4	0.1–1.9
Comorbidities						
Cancer	1 (0)			3 (0)		
Food allergy	14 (2)			35 (7)		
Immune disorder^c^	1 (0)			6 (33)	**8.4**	**1.5–47**
Asthma	2 (100)	n.c.		12 (27)	**8.1**	**2.3–29**

^a^Standardized by gender, age (five-year age groups) and location of residence (urban, intermediate, rural).

^b^Day-care, kindergarten or guest parent attendance of other children in the household

^c^E.g. Guillain Barre Syndrome, Grave’s disease, Addison’s disease, sarcoidosis

GE: gastroenteritis, OR: odds ratio, 95% CI: 95% confidence interval, n.c.: non calculable

In (young) adults in the age group 18–24 years, risk factors were living in a household with children aged 3–12 years (OR 25.6, 95%CI: 3.9–168), compared with living in a household without children (Table 3). In the age group 25–44 years, those living in a household with a child aged 3–12 years and at least one child attending out-of-home care, had more GE than those living in a household without children (OR 2.8, 95%CI 1.1–7.3). Non-native Dutch males also had more GE compared to native Dutch males (OR 4.0, 95%CI: 1.1–15). In the age groups 25–44 years and 45–64 years, employed persons with a high educational level had fewer episodes of GE compared with those unemployed (OR 0.4, 95%CI: 0.1–0.9 and OR 0.4, 95%CI: 0.2–0.9, respectively). In the age group 45–64 years, the same was observed for employed persons with an intermediate educational level compared with unemployed persons (OR 0.5, 95%CI: 0.3–1.0).

**Table 3:** Multivariable weighted logistic regression models of factors associated with gastroenteritis in adults, stratified by age groups.

	18–24 years (n = 555)			25–44 years (n = 1,784)			45–64 years (n = 2,892)			65+ years (n = 1,874)		
	N (%GE)	OR^a^	95%CI	N (%GE)	OR^a^	95%CI	N (%GE)	OR^a^	95%CI	N (%GE)	OR^a^	95%CI
Children in household												
No children	411 (11)	Ref		758 (6)			2,262 (5)	Ref		1,863 (3)	-	
0y	1 (0)	n.c.		61 (13)			5 (0)	n.c.		0		
1-2y	2 (0)	n.c.		86 (7)			5 (0)	n.c.		0		
3-12y	7 (68)	**25.6**	**3.9–168**	470 (6)			123 (6)	1.2	0.5–2.8	1 (0)		
13-17y	109 (12)	1.4	0.5–3.9	80 (2)			507 (3)	0.6	0.3–1.2	4 (0)		
Children x daycare^b^												
No children	411 (11)	-		758 (6)	Ref		2,262 (5)	-		1,863 (3)	-	
No daycare, 0y	0			28 (14)	3.7	0.4–32	4 (0)			0		
Daycare, 0y	1 (0)			33 (11)	3.0	0.5–19	1 (0)			0		
No daycare, 1-2y	1 (0)			22 (4)	0.9	0.1–6.3	3 (0)			0		
Daycare, 1-2y	1 (0)			64 (8)	1.8	0.6–5.3	2 (0)			0		
No daycare, 3-12y	6 (78)			310 (4)	0.7	0.2–2.1	100 (6)			1 (0)		
Daycare, 3-12y	1 (0)			160 (10)	**2.8**	**1.1–7.3**	23 (7)			0		
13-17y	109 (12)			80 (2)	0.2	0.0–1.3	507 (3)			4 (0)		
Gender x migration^d^												
Dutch male	214 (11)	-		700 (5)	Ref		1,308 (4)	-		977 (2)	Ref	
Non-Dutch male	9 (11)			50 (16)	**4.0**	**1.1–15**	70 (1)			70 (1)	0.4	0.1–3.5
Dutch female	324 (12)			981 (7)	1.1	0.6–2.3	1,587 (5)			768 (4)	**2.1**	**1.1–4.2**
Non-Dutch female	8 (16)			53 (3)	0.3	0.0–2.2	117 (11)			59 (13)	**6.4**	**2.5–17**
Employment x education												
No employment	225 (6)	Ref		229 (8)	Ref		803 (6)	Ref		1,580 (3)	Ref	
Yes, low	44 (5)	0.5	0.1–2.8	194 (6)	0.8	0.2–2.5	615 (5)	0.9	0.5–1.7	24 (13)	**4.6**	**1.1–20**
Yes, intermediate	198 (15)	1.7	0.6–4.8	536 (6)	0.5	0.2–1.2	755 (3)	**0.5**	**0.3–1.0**	14 (0)	n.c.	
Yes, high	73 (12)	3.1	0.9–10	784 (6)	**0.4**	**0.1–0.9**	795 (3)	**0.4**	**0.2–0.9**	22 (0)	0.9	0.1–6.8
Comorbidities												
Cancer	0	-		18 (10)	-		114 (1)	-		137 (9)	**3.0**	**1.4–6.5**
Food allergy	28 (24)	-		68 (5)	-		64 (5)	-		29 (14)	**3.5**	**1.3–9.3**
Immune disorder^c^	10 (11)	-		26 (9)	-		42 (5)	-		22 (0)	-	
Asthma	5 (26)	-		12 (0)	-		18 (9)	-		0	-	

^a^Standardized by gender, age (five-year age groups) and location of residence (urban, intermediate, rural).

^b^Day-care, kindergarten or guest parent attendance of at least one child in the household

^c^E.g. Guillain Barre Syndrome, Grave’s disease, Addison’s disease, sarcoidosis

^d^Born in- or outside the Netherlands

GE: gastroenteritis, OR: odds ratio, 95% CI: 95% confidence interval, n.c.: non calculable

In persons 65 years or older, females with and without native Dutch ethnic background had more GE compared with native Dutch males (OR 6.4, 95%CI: 2.5–17 and OR 2.1, 95%CI: 1.1–4.2). Those with a food allergy (OR 3.1, 95%CI: 1.2–8.4) or cancer (OR 3.1, 95%CI: 1.5–6.4) also had more GE. Employed persons with a low educational level had more GE compared with unemployed or retired persons (OR 4.6, 95%CI: 1.1.-20).

### Costs per gastroenteritis episode

An estimated 123/544 GE cases (6.3%, standardized: 6.4%) visited the GP at least once, with an average of 0.13 visits per GE episode (standardized: 0.12 visits), and 9/544 cases required hospitalization (1.7%, standardized: 1.6%). Seven cases (1.7%, standardized: 1.3%) reported that stool sample tests were performed (Table 4). Medicines were prescribed by a doctor in 43 cases (8.7%, standardized: 7.8%) and bought OTC by 193 cases (35.8%, standardized: 35.3%).

**Table 4:** Average use of resources per gastroenteritis case, by age group.

Average use per GE case	0–4 (n = 522)	5–17 (n = 1,439)	18–24 (n = 555)	25–44 (n = 1,784)	45–64 (n = 3,082)	65+ (n = 1,874)	All ages (n = 9,256)
**Direct healthcare costs**							
GP visit	0.13	0.06	0.07	0.12	0.11	0.26	0.12
Hospitalization days	0.07	0.03	0.00	0.05	0.12	0.83	0.14
Medicine prescription	0.17	0.02	0.08	0.09	0.09	0.15	0.09
Stool sample laboratory test	0.03	0.01	0.01	0.00	0.03	0.00	0.01
**Patient costs**							
Car//bus transport	0.10	0.06	0.07	0.12	0.11	0.26	0.12
Ambulance transport	<0.01	<0.01	0.00	<0.01	<0.01	0.05	0.01
Medicine OTC	0.29	0.40	0.44	0.44	0.49	0.36	0.42
**Productivity losses**							
Hours absence paid work^a^	2	1	4.5	4.5	4.5	0.2	3.0

^a^Of themselves or a caregiver

GE: gastroenteritis, GP: general practitioner, OTC: over-the-counter

The average costs per GE episode was €126 in children 0–4 years, €61 in the age group 5–17 years, €82 in the age group 18–24 years, €182 in the age group 25–44 years, €261 in the age group 45–64 years, and €475 in persons ≥65 years (Table 5). The majority of overall costs were due to absence from paid work (55%) and hospitalization (36%). Productivity losses (i.e. the costs due to absence from paid work) were highest in the age group 40–65 years and hospitalization costs were highest in those ≥65 years. Patient costs accounted for only 2% of the overall costs.

**Table 5:** Average costs of gastroenteritis by resource unit and age group, and estimated total costs for the Netherlands.

Average costs per GE case (€)	0–4	5–17	18–24	25–44	45–64	65+	All ages
**Direct healthcare costs**							
GP visit	6.22	2.68	3.28	5.19	4.79	10.85	5.18
Hospitalization	46.21	21.33	0.00	24.72	60.81	403.52	68.45
Medicine prescription	1.31	0.14	0.35	0.64	0.75	1.21	0.69
Stool sample laboratory test	1.94	0.89	0.41	0.00	2.56	14.55	0.98
Total	55.68	25.04	4.04	30.55	68.91	430.14	80.80
**Patient costs**							
Car/bus transport	0.30	0.14	0.13	0.24	0.25	0.58	0.24
Ambulance transport	0.16	0.07	0.00	0.61	1.50	30.00	3.59
Medicine OTC	0.40	0.56	0.60	0.60	0.68	0.49	0.58
Total	0.86	0.77	0.73	1.46	2.43	31.07	4.40
**Productivity losses**							
Absence paid work^a^	69.50	34.75	77.33	149.83	189.97	13.90	105.10
**Average costs per GE episode**	126.04	60.56	82.10	181.83	261.31	475.11	190.70
**Total costs Dutch population**							
Population size	872,500	2,532,390	1,479,740	4,215,470	4,824,970	3,278,160	17,203,230
Total annual costs (millions)	77.28	44.63	64.18	231.88	270.06	256.52	944.56
Average annual costs per person	88.57	17.62	43.37	55.01	55.97	78.25	54.91
With children in the household	88.57	17.62	49.55	59.51	42.80	n/a	53.38^b^
Without children in the household	n/a	n/a	41.09	50.88	61.09	78.25	54.85^b^

^a^Of themselves or a caregiver

^b^Only persons 18–64 years living with and without children in the household

GE: gastroenteritis, GP: general practitioner, OTC: over-the-counter

With overall average costs per GE episode of €191 and an adjusted incidence of 0.29 episodes/person-year, the estimated costs of GE for the Netherlands in 2017 amounted to €945 million. Without correction for potential overestimation of our incidence, the upper boundary of the costs would be €2.6 billion. When averaging the total costs over the total number of inhabitants, the costs were €55 per inhabitant per year (€945 million/17.2 million inhabitants), and €154 as upper boundary (€2.6 billion/17.2 million inhabitants). The average costs per inhabitant aged 18–64 years were similar for those living with and without children in the household, namely €53 and €55, respectively. However, differences were observed when further stratifying by age. In the age groups 18–24 years and 25–44 years, costs were higher in those living with children in the household than those living without children in the household (18–24 years: €50 and €41, respectively; 25–44 years: €60 and €51, respectively). However, in the age group 45–64 years, costs were lower in those living with children in the household than those living without children in the household (€43 and €61, respectively).

## Discussion

This study provides updated estimates of the incidence of GE in different strata of the Dutch community and the first estimates of such incidence in households with and without children. The overall incidence was estimated at 0.81 GE episodes per person-year, with the highest incidence in children aged 0–4 years and the lowest one in those aged ≥65 years. The average costs per GE episode were estimated at €191, corresponding to a total economic burden of €945 million in 2017. Without correction for potential overestimation of the GE incidence, the costs would be €2.6 billion.

The observed GE incidence was slightly lower than the previous estimate of 0.96 GE episodes per person-year in the Netherlands in 2009 [2]. In general, retrospective studies in other European countries have found higher GE incidence rates, namely 0.9 GE episodes/person-year in Poland, 1.1 GE episodes/person-year in Italy, and 1.4 GE episodes/person-year in Denmark [15, 16, 18]. The incidence was, however, substantially higher than estimates from France, with 0.3 GE episodes/person-year during 2009–2010 [20]. In the latter study authors attributed the low estimate to a higher exclusion rate of GE episodes likely due to non-infectious causes. Indeed, they excluded 46% of GE episodes, compared with 15% in our study. Of note, data collection in these studies were performed through telephone interviews, while we performed a web-based survey. The major advantage of a web-based survey is that it is less time-consuming, but a compromise is the response rate, which was 20% in our survey compared to 26–81% in the telephone surveys, which might affect the representativeness of our sample [15, 16, 18, 20]. Moreover, while our design required participants to have a computer with internet access, and a basic level of computer skills, telephone surveys require persons to have a landline and/or a registered mobile phone number in national phonebooks (unless they can be obtained from mobile network providers). Hence, both study designs might reach different populations. Although comparison of incidence estimates of community-acquired GE by other countries is hampered by the fact that they used different case definitions, estimates varied between 0.4 and 1.4 episodes/person-year: 0.4 episodes/person-year in Malta in 2004–2005, 0.6 episodes/person-year in Ireland in 2000–2001, 0.9 episodes/person-year in Australia in 2001–2002 1.2 episodes/person-year in Norway in 1999–2000, 1.2 episodes/person-year in Canada in 2005–2006, and 1.4 episodes/person-year in the United States of America in 1996–1997 [17, 19, 30–34].

The observed incidence of 0.81 episodes/person-year is likely an overestimation of the true incidence due to the retrospective nature of the study, which is prone to respondents attributing GE episodes that occurred earlier to the survey period (i.e. ‘telescoping’), as well as selection bias of persons who recently experienced GE. Indeed, prospective studies tend to produce lower GE estimates, as was observed in a prospective population-based cohort studies from the Netherlands in 1999 and the United Kingdom in 2009, with 0.28 episodes/person-year and 0.27 episodes/person-year, respectively [7, 21]. In the latter study, they found a three-fold higher GE estimate in a retrospective study that was conducted in parallel to the prospective study in the same study population [21]. When applying the same correction factor to our GE incidence estimate, we would obtain an incidence of 0.29 episodes/person-year. This would suggest that the incidence of GE had remained at the same level as 20 years ago. Importantly, also prospective studies are subject to biases, including selection bias [22]. Hence, although the corrected incidence may provide an approximation of the true incidence, it should be interpreted with caution.

In children aged 0–4 years, living in a household with other children of less than 1 year of age was a risk factor for GE. This is most likely due to increased transmission between siblings [9, 10]. Interestingly, asthmatic children in this age group also had more GE, which is in line with findings from the previous study on GE in the Dutch community [2]. The hypothesis of asthmatic individuals being generally frailer and thereby also more sensitive to GE has been proposed before, but the mechanism remains largely unclear [35, 36]. In children aged 5–17 years, those with an immune disorder were more likely to have GE. Indeed, immune-related diseases are associated with altered regulatory mechanisms between active immunity and tolerance in the gut, rendering these children more vulnerable to gastrointestinal infections [37].

In persons aged 18–24 and 25–44 years, having children aged 3–12 years in the household was also a risk factor for GE, especially if these children attended out-of-home care, likely reflecting transmission from young children to their parents [9, 10]. Interestingly, having children in the household was not a risk factor in persons aged 45–64 years. Indeed, these children were generally older and required less hands-on care, thereby likely reducing opportunities for transmission. A previous Dutch study among families with preschool children also found that GE risk of parents decreased with parents’ age [9]. In the age group 25–44 years, non-native Dutch males more often had GE. Although we do not have a clear explanation, a community-based study in Italy found a similar higher GE incidence among non-Italian citizens [16]. Intermediate and highly educated persons in the age groups of 25–44 years and 45–64 years who were employed had GE less often than unemployed persons in the same age group. We hypothesize two possible reasons. First, the unemployed may reflect a population that is more vulnerable to infection, as health complaints might be the reason for unemployment, and second, they might be unemployed to take care of their children, which in itself could be a risk factor for GE. In contrast, being employed was a risk factor for GE in the age group of ≥65 years, which we believe reflects the ‘younger elders’ that are somewhat more socially active among the elderly. Indeed, three out of four cases with GE cases in this age group were 65–69 years. In the age group of ≥65 years, females more often had GE compared with native Dutch males. Although gender differences have been reported by other countries as well, albeit not always significant, they generally observed younger adult females to be at increased risk for GE, but not females aged ≥65 years [15, 17, 18]. Our finding is in agreement with a survey from the Netherlands, however, reporting that 26% of woman help caring for their grandchildren compared with 20% in males [38]. Furthermore, food allergy was a risk factor for GE in those 65+ years, for which the explanation could be bi-directional. While gastrointestinal infections have been identified as risk factor for food allergy, food allergy can reversely be the cause of diarrhea [39]. Moreover, specific changes to the immune system in some immune deficiency diseases may increase the risk of the developing food allergies, acting as a risk factor for GE [39].

We estimated the average costs of a GE episode in the Netherlands to be €191, which gives a total of €945 million costs for GE in the whole country in 2017 (= €55 per inhabitant). This is higher than previous estimates of €133–151 per GE episode (€150–170 after correcting for inflation) amounting to €611–695 million (€687–782 after correction) of total costs (€37–42 per inhabitant; €42–47 after correction) in 2009 [3]. This increase is partially attributable to increased healthcare costs. For example, costs for GP practice visits and hospitalization have increased by 18% and 7%, respectively, between 2009 and 2017. However, increased costs are mainly due to a higher hospitalization rate of GE cases in our study: 1.9% *vs*. 0.3% reported previously [3, 4],. It might be that our hospitalization rate is an overestimation due to selection bias of those who were recently hospitalized. The previous estimate was based on data from a prospective study on GE, which is less prone to this type of bias. Comparing our results with COI studies on GE from other countries is difficult due to differences in healthcare systems [17, 32, 40, 41]. However, in line with our findings, most studies report the major factor determining the magnitude of estimated economic costs to be productivity losses by the sick and/or their caregivers.

In contrast to the previous study estimating the GE incidence in the Dutch community in 2009, we could not exclude cases with vomiting due to regurgitation, motion sickness/vertigo, nauseous event, traumatic event, pregnancy, or alcohol/drug abuse, because these data were not available. Of the GE cases in our study, 13% had diarrhea and vomiting, 47% had diarrhea without vomiting, and 40% had vomiting without diarrhea. This differs from the 2009 study in the Netherlands, where 19% had both, 55% only diarrhea, and 26% only vomiting. The proportion of excluded GE episodes due to likely non-infectious causes in our study (15%) was similar to the one of the 2009 study (18%) [2]. Although some misclassification is likely to have occurred, this cannot fully explain the difference. A large proportion (43%) of GE cases reporting vomiting only, were children younger than 15 years, which is unlikely to be the result of alcohol abuse or pregnancy. Moreover, we have no indication for misclassification of vomiting due to regurgitation. In the age group 0–4 years, where regurgitation is most likely to occur, the incidence was even lower in our study than the previous one, i.e., 1.96 episodes/person-years compared with 2.89 episodes/person-year, respectively. This could be due to the exceptionally low rotavirus activity observed during 2014–2016 in the Netherlands, in the absence of rotavirus vaccination as part of the national immunization programme, which is the most important cause of GE in children in this age group [42]. The annual number of laboratory confirmed rotavirus detections in the Netherlands ranged from 607 to 1323 during 2014–2016, compared with1936 rotavirus detections in 2009. Of note, rotavirus vaccination is scheduled to be implemented as part of the immunization programme in the Netherlands in 2019, but only for children with medical risk conditions predisposing to severe or complicated rotavirus GE, including prematurity, low birth weight, and severe congenital pathology [43, 44].

Several additional limitations should be recognised. The survey did not collect data on all parameters that were needed to calculate costs. Hence, we relied on existing data from the available literature to base our assumptions on. For example, we based the average duration of hospitalization on a study from 2009, while the current duration is likely to be shorter due to ongoing changes in Dutch hospital policy. Also, in order to ensure comparability with the previous study on GE costs in the Netherlands, we did not stratify productivity losses by mild, moderate (GP visits) and severe (hospitalized) cases. This could have led to an underestimation of the costs, since the hospitalization rate in our study was higher, which is associated with higher productivity losses. Moreover, it is crucial to consider the characteristics of respondents *vs*. those that did not participate. We adjusted (statistical weighting) for deviations of the sample from the underlying population it was drawn from, including an adjustment of the number of GP visits in our survey based on independently recorded GP surveillance data. Yet, our estimates represent the lower boundary of the true value of costs due to GE. First, we had no information (and could not therefore include data) on premature mortality and chronic sequelae due to GE, while previous research has shown that these generally dominate the estimates of costs associated with GE morbidity [24]. Second, we had no information on costs associated with productivity losses due to non-paid employment work (e.g. child care, voluntary work). And third, we considered only the direct effect of GE, and ignored potential sequelae triggered by GE infections [24, 28].

In conclusion, GE still poses a significant burden in the community, particularly in children of 0–4 years of age and in adults of ≤44 years of age living in households with children, and especially if children attend out-of-home childcare services. Similar to other industrialized countries, the major factor driving the costs due to GE was the loss of productivity (paid worktime) for the ill or the caregiver(s) of the ill. This study also provides new baseline GE incidence rates and characterizes the population at greatest risk. Moreover, the estimated annuals costs of GE can be used by public health policy makers to contextualize the economic burden of the disease in support of decision making.

## Supporting information

S1 Questionnaire ESBLAT study questionnaire in Dutch. (DOC)

S2 Questionnaire ESBLAT study questionnaire in English. (DOC)

S1 Table Unit costs in Euros, 2017. (DOCX)

S2 Table Population characteristics of the ESBLAT study population (before and after weighting) and the Dutch population. (DOCX)
