# Supplementary material for: Incidence and economic burden of community-acquired gastroenteritis in the Netherlands: Does having children in the household make a difference?
Source: PLoS One. 2019 May 23;14(5):e0217347. doi: 10.1371/journal.pone.0217347 (PMC6532970; doi:10.1371/journal.pone.0217347)
Supplement: S1 Questionnaire — (DOC) [file pone.0217347.s001.doc]

Bevolkingsonderzoek naar antibiotica-resistentie

| **Bevolkingsonderzoek naar antibiotica-resistentie** |
| --- |

| Welkom!  Deelname aan dit onderzoek is geheel vrijwillig. Als u deelneemt heeft u recht om uw toestemming op ieder moment weer in te trekken zonder dat u daar een reden voor op hoeft te geven.  Bij vragen en/of problemen kunt u telefonisch of (bij voorkeur) per e-mail contact opnemen met de onderzoekers van het UMCU en het RIVM. U vindt de contactgegevens van de onderzoekers in de brief die u heeft ontvangen.  U gegevens zullen anoniem blijven en niet aan derden worden verstrekt.  Bij voorbaat hartelijk dank voor uw medewerking! |
| --- |

| **Dit zijn de onderwerpen waar we vragen over gaan stellen. Afhankelijk van uw antwoorden, zullen sommige delen sneller klaar zijn dan andere.** |
| --- |

| * Algemene vragen  * Gezin en huishouden  * Gezondheid  * Antibioticagebruik  * Gezondheidsklachten  * Opleiding en werk  * Contact met dieren en vlees  * Voeding  * Hygiëne  * Reizen en andere activiteiten  * Laboratoriumonderzoek  * Huisdiervragen  Aan het einde vragen we uw toestemming om uw antwoorden te gebruiken voor ons onderzoek. Afhankelijk van de leeftijd van de uitgenodigde persoon hebben we ook toestemming nodig van de ouder(s)/verzorger(s). |
| --- |

|  |
| --- |

| **ALGEMENE VRAGEN** |
| --- |

| **1. Wordt deze vragenlijst ingevuld door de uitgenodigde persoon zelf? (met geboortedatum [Geboortedatum])** |
| --- |

| ❑ | Nee |
| --- | --- |
| ❑ | Ja |

|  |
| --- |

| **2. Wat is uw relatie met de uitgenodigde persoon?** |
| --- |

| Wilt u de vragen invullen zodat het klopt voor de uitgenodigde persoon? |
| --- |

| ❑ | Ouder/verzorger |
| --- | --- |
| ❑ | Broer/zus |
| ❑ | Zoon/dochter |
| ❑ | Partner |
| ❑ | Anders namelijk ___________ |

|  |
| --- |

| **3. Is de geboortedatum van de uitgenodigde persoon:**  **[Geboortedatum]** |
| --- |

| ❑ | Dit klopt |
| --- | --- |
| ❑ | Dit klopt niet. Het moet zijn: ___________ |

| **4. Wat is uw geboorteland?** |
| --- |

| (Staat uw geboorteland niet in de lijst, kiest u dan onderaan de optie 'Anders') | |
| --- | --- |
|  |  |

| **5. Mijn geboorteland is:** |
| --- |

| ______________________________ |
| --- |

|  |
| --- |

| **GEZIN EN HUISHOUDEN** |
| --- |

| De volgende vragen gaan over de samenstelling en gebruiken van uw huishouden (het huishouden van de persoon met [Geboortedatum] als geboortedatum) |
| --- |

| **6. Uit hoeveel (thuiswonende) personen bestaat uw huishouden?** |
| --- |

| (uzelf/geadresseerde meegerekend) |
| --- |

| ❑ | 1 |
| --- | --- |
| ❑ | 2 |
| ❑ | 3 |
| ❑ | 4 |
| ❑ | 5 |
| ❑ | 6 |
| ❑ | 7 |
| ❑ | 8 |
| ❑ | 9 |
| ❑ | 10 |
| ❑ | Meer dan 10 |

| **7. Wat is uw woonsituatie?** |
| --- |

| ❑ | Ik woon in een eengezinswoning (tussenwoning, 2-onder-1-kapwoning, vrijstaande woning) |
| --- | --- |
| ❑ | Ik woon in een appartement |
| ❑ | Ik woon in een studentenwoning |
| ❑ | Ik woon in een verpleeg- of verzorgingstehuis |
| ❑ | Anders dan bovenstaande, namelijk: ___________ |
| ❑ | Ik woon in een boerderij |

| **8. Zijn er (thuiswonende) kinderen in uw huishouden?** |
| --- |

| ❑ | Nee |
| --- | --- |
| ❑ | Ja |

|  |
| --- |

| **9. Uit hoeveel (thuiswonende) kinderen bestaat uw huishouden?** |
| --- |

| ❑ | 1 |
| --- | --- |
| ❑ | 2 |
| ❑ | 3 |
| ❑ | 4 |
| ❑ | 5 |
| ❑ | 6 |
| ❑ | 7 |
| ❑ | 8 |
| ❑ | Meer dan 8 |

| **10. Hoe oud zijn de (thuiswonende) kinderen in uw huishouden?** |
| --- |

| Meerdere antwoorden mogelijk |
| --- |

| ❑ | 0 t/m 12 maanden |
| --- | --- |
| ❑ | Tussen 1 en 2 jaar |
| ❑ | 3 t/m 12 jaar |
| ❑ | 13 t/m 18 jaar |
| ❑ | Ouder dan 18 jaar |

|  |
| --- |

| **11. Zijn er binnen uw huishouden kinderen die naar een kinderdagverblijf gaan?** |
| --- |

| (meerder antwoorden mogelijk) |
| --- |

| ❑ | Nee |
| --- | --- |
| ❑ | Ja, de uitgenodigde zelf |
| ❑ | Ja, een kind uit het huishouden |

| **12. Zijn er binnen uw huishouden kinderen die naar een peuterspeelzaal gaan?** |
| --- |

| (meerdere antwoorden mogelijk) |
| --- |

| ❑ | Nee |
| --- | --- |
| ❑ | Ja, de uitgenodigde zelf |
| ❑ | Ja, een kind uit het huishouden |

| **13. Zijn er binnen uw huishouden kinderen die naar een gastouder gaan?** |
| --- |

| (meerdere antwoorden mogelijk) |
| --- |

| ❑ | Nee |
| --- | --- |
| ❑ | Ja, de uitgenodigde zelf |
| ❑ | Ja, een kind uit het huishouden |

| **14. Hoeveel kinderen in uw huishouden dragen luiers?** |
| --- |

| ❑ | 0 |
| --- | --- |
| ❑ | 1 |
| ❑ | 2 |
| ❑ | 3 |
| ❑ | Meer dan 3 |

|  |
| --- |

| **15. Wordt er in uw huishouden gerookt?** |
| --- |

| ❑ | Nee |
| --- | --- |
| ❑ | Ja, buitenshuis |
| ❑ | Ja, binnenshuis |
| ❑ | Weet ik niet |

| **16. Welke van de onderstaande opties is van toepassing op u?** |
| --- |

| (meerdere antwoordopties mogelijk) |
| --- |

| ❑ | Mijn huis en/of werkadres ligt aan een bosrand |
| --- | --- |
| ❑ | Mijn huis en/of werkadres ligt bij een stadspark/stadsbos |
| ❑ | Mijn huis en/of werkadres ligt bij een weiland |
| ❑ | Mijn huis en/of werkadres ligt bij een akkerland |
| ❑ | Mijn huis en/of werkadres ligt aan het water (zee, meer, rivier, beek, kanaal, etc.) |
| ❑ | Geen van bovenstaande |

|  |
| --- |

| **GEZONDHEID** |
| --- |

| Hieronder volgen vragen over uw gezondheid (de persoon met geboortedatum [Geboortedatum]) |
| --- |

| **17. Bent u de afgelopen 12 maanden opgenomen in een zorginstelling? (1 overnachting of meer)** |
| --- |

| (Meerdere antwoorden mogelijk) |
| --- |

| ❑ | Nee |
| --- | --- |
| ❑ | Ja, in een ziekenhuis |
| ❑ | Ja, in een verpleeghuis |
| ❑ | Ja, in een verzorgingstehuis |
| ❑ | Ja, in een revalidatiecentrum |
| ❑ | Ja, anders namelijk ___________ |

|  |
| --- |

| **18. Hoeveel keer bent u de afgelopen 12 maanden opgenomen?** |
| --- |

| (1 overnachting of meer) |
| --- |

| ❑ | 1 |
| --- | --- |
| ❑ | 2 |
| ❑ | 3 |
| ❑ | 4 |
| ❑ | 5 |
| ❑ | 6 |
| ❑ | 7 |
| ❑ | 8 |
| ❑ | 9 |
| ❑ | 10 |
| ❑ | Meer dan 10 keer |

| **19. Was/waren de opname(s) in het buitenland?** |
| --- |

| ❑ | Dit was in Nederland |
| --- | --- |
| ❑ | Dit was in het buitenland |

|  |
| --- |

| **20. In welk(e) land(en) bent u opgenomen?** |
| --- |

| (meerdere antwoorden mogelijk) | |
| --- | --- |
|  |  |

| **21. Op wat voor een soort afdeling bent u opgenomen?** |
| --- |

| (meerdere antwoorden mogelijk) |
| --- |

| ❑ | Op een korte verblijf (short-stay afdeling) |
| --- | --- |
| ❑ | Op een algemene verpleegafdeling (bijvoorbeeld oncologie-, urologie-, chirurgie-, interne-, nefrologie-, long-, reuma-, maag-, darm en lever-, cardiologieafdeling, etc.) |
| ❑ | Op een intensive care afdeling (IC) of hartbewakingsafdeling (CCU) |
| ❑ | Anders, namelijk ___________ |

|  |
| --- |

| **22. Is één van de andere (thuiswonende) gezinsleden afgelopen 12 maanden opgenomen (1 overnachting of meer)?** |
| --- |

| (Meerdere antwoorden mogelijk) |
| --- |

| ❑ | Nee |
| --- | --- |
| ❑ | Ja, in een ziekenhuis |
| ❑ | Ja, in een verpleeghuis |
| ❑ | Ja, in een verzorgingstehuis |
| ❑ | Ja, in een revalidatiecentrum |
| ❑ | Ja, anders namelijk ___________ |

|  |
| --- |

| **23. Welke langdurige gezondheidsklachten en/of ziekten zijn op u van toepassing (met geboortedatum [Geboortedatum])** |
| --- |

| (meerdere antwoorden mogelijk) |
| --- |

| ❑ | Ziekte van het maagdarmkanaal |
| --- | --- |
| ❑ | Voedselallergie |
| ❑ | Diabetes (suikerziekte) |
| ❑ | Afwezigheid van de milt |
| ❑ | Leverziekte |
| ❑ | Nierziekte |
| ❑ | Hart- en /of vaatziekten |
| ❑ | Kanker |
| ❑ | Immuunstoornis |
| ❑ | Reuma |
| ❑ | Longziekte |
| ❑ | Anders, namelijk ___________ |
| ❑ | Geen klachten |

| **24. Heeft u de afgelopen 6 maanden medicijnen op doktersrecept gehad?** |
| --- |

| (Meerdere antwoorden mogelijk) |
| --- |

| ❑ | Nee |
| --- | --- |
| ❑ | Ja, medicijnen vanwege een infectie (bijvoorbeeld antibiotica) |
| ❑ | Ja, maagzuurremmers (zoals bijv. omeprazol, pantoprazol, lansoprazol, rabeprazol, esomeprazol) |
| ❑ | Ja, cholesterolverlagers (zoals bijv. simvastatine, pravastatine, atorvastatine) |
| ❑ | Ja, laxeermiddelen (zoals bijv. colofort, endofalk, movicolon, molaxole) |
| ❑ | Ja, bloeddrukverlagende middelen (zoals betaxolol, valsartan, amlodipine, enz.) |
| ❑ | Ja, ADHD medicatie (zoals ritalin) |
| ❑ | Ja, middelen tegen depressie (zoals venlafaxine, Lithium, MAO-remmers, enz.) |
| ❑ | Ja, slaap- en kalmeringsmiddelen (zoals diazepam, oxazepam, temazepam, enz.) |
| ❑ | Ja, antidiabetica (zoals metformine, glimepiride, levemir, novorapid, enz.) |
| ❑ | Ja, chemotherapie |
| ❑ | Ja, anticonceptiepil (alleen voor vrouwen) |
| ❑ | Ja, anders namelijk ___________ |

|  |
| --- |

| **ANTIBIOTICAGEBRUIK** |
| --- |

| Antibiotica worden voorgeschreven voor behandeling van bepaalde infecties. De arts schrijft een antibioticum voor in een bepaalde hoeveelheid en voor een bepaald aantal dagen. Dit wordt een antibioticumkuur genoemd.  Voorbeelden van antibiotica zijn penicilline (Penidural®), feneticilline (Broxil®), flucloxacilline (Floxapen®), amoxicilline, amoxicilline-clavulaanzuur (Augmentin®), nitrofurantoïne (Furadantine®, Furabid®), doxycycline, azitromycine (Zithromax®) en ciprofloxacine (Ciproxin®). Het kan best zijn dat u antibiotica heeft gebruikt of kent die hier niet tussen staan. Er zijn namelijk nog veel meer antibiotica op de markt.    LET OP! Onderstaande vragen gaan over de persoon aangeschreven in de brief (met als geboortedatum [Geboortedatum]) |
| --- |

| **25. Wanneer heeft u voor het laatst antibiotica gebruikt?** |
| --- |

| ❑ | Nooit |
| --- | --- |
| ❑ | De laatste keer was minder dan een maand geleden |
| ❑ | De laatste keer was 1 tot 3 maanden geleden |
| ❑ | De laatste keer was 4 tot 6 maanden geleden |
| ❑ | De laatste keer was 6 maanden tot een jaar geleden |
| ❑ | De laatste keer was meer dan een jaar geleden |

|  |
| --- |

| **26. Hoe vaak heeft u de afgelopen 12 maanden een antibioticumkuur gebruikt?** |
| --- |

| (Indien een kuur verlengd is, kunt u de totale kuur als 1 keer beschouwen) |
| --- |

| ❑ | 1 keer |
| --- | --- |
| ❑ | 2 keer |
| ❑ | 3 keer |
| ❑ | 4 keer |
| ❑ | 5 keer |
| ❑ | Meer dan 5 keer |

|  |
| --- |

| **27. Wat was de naam van het laatst gebruikte antibioticum?** |
| --- |

| ❑ | penicilline (Penidural®) |
| --- | --- |
| ❑ | feneticilline (Broxil®) |
| ❑ | flucloxacilline (Floxapen®) |
| ❑ | amoxicilline |
| ❑ | amoxicilline-clavulaanzuur (Augmentin®) |
| ❑ | nitrofurantoïne (Furadantine®, Furabid®) |
| ❑ | doxycycline |
| ❑ | azitromycine (Zithromax®) |
| ❑ | ciprofloxacine (Ciproxin®) |
| ❑ | ceftriaxon |
| ❑ | Anders, namelijk ___________ |
| ❑ | Weet ik niet |

| **28. Wat was de dosering van dit antibioticum (per inname keer) in mg?** |
| --- |

| ❑ | 50 mg |
| --- | --- |
| ❑ | 100 mg |
| ❑ | 200 mg |
| ❑ | 250 mg |
| ❑ | 300 mg |
| ❑ | 400 mg |
| ❑ | 500 mg |
| ❑ | 600 mg |
| ❑ | Anders, namelijk ___________ |
| ❑ | Weet ik niet |

| **29. Hoeveel dagen heeft u de laatste antibioticumkuur, die u voorgeschreven kreeg, gebruikt?** |
| --- |

| ❑ | 1 |
| --- | --- |
| ❑ | 2 |
| ❑ | 3 |
| ❑ | 4 |
| ❑ | 5 |
| ❑ | 6 |
| ❑ | 7 |
| ❑ | 8 |
| ❑ | 9 |
| ❑ | 10 |
| ❑ | 11 |
| ❑ | 12 |
| ❑ | 13 |
| ❑ | 14 |
| ❑ | Meer dan 14 |
| ❑ | Weet ik niet |

| **30. Hoeveel keer per dag nam u uw laatste antibioticumkuur?** |
| --- |

| ❑ | 1 |
| --- | --- |
| ❑ | 2 |
| ❑ | 3 |
| ❑ | 4 |
| ❑ | Meer dan 4 keer |
| ❑ | Weet ik niet |

| **31. Voor welke infectie heeft u de laatste antibioticumkuur voorgeschreven gekregen?** |
| --- |

| ❑ | Urineweginfectie (blaasontsteking) |
| --- | --- |
| ❑ | Longontsteking |
| ❑ | Keelontsteking |
| ❑ | Maagdarminfectie |
| ❑ | Anders, namelijk ___________ |

|  |
| --- |

| **32. Hoeveel keer heeft u de afgelopen 6 maanden een blaasontsteking gehad?** |
| --- |

| ❑ | 1 |
| --- | --- |
| ❑ | 2 |
| ❑ | 3 |
| ❑ | 4 |
| ❑ | Meer dan 4 |

|  |
| --- |

| **33. Hoe heeft u de laatste antibioticumkuur verkregen?** |
| --- |

| ❑ | Op recept van de huisarts |
| --- | --- |
| ❑ | Op recept van de behandelend arts in het ziekenhuis |
| ❑ | Op recept van tandarts |
| ❑ | Op recept via het reizigersspreekuur |
| ❑ | Op recept van kennis/familielid |
| ❑ | Via internet zonder recept |
| ❑ | In het buitenland zonder recept |
| ❑ | Van een restje van iemand anders |
| ❑ | Anders, namelijk ___________ |

| **34. Is bij de laatste antibioticumkuur aan u verteld hoe u het antibioticum moet gebruiken?** |
| --- |

| (meerdere antwoorden mogelijk) |
| --- |

| ❑ | Nee |
| --- | --- |
| ❑ | Ja, door de huisarts of assistente van de huisarts |
| ❑ | Ja, door de behandelend arts of assistente in het ziekenhuis |
| ❑ | Ja, door de tandarts of tandartsassistente |
| ❑ | Ja, door de apotheker of apothekersassistente |
| ❑ | Ja, door iemand anders, namelijk ___________ |

|  |
| --- |

| **35. Heeft u de laatste antibioticumkuur volgens de bijsluiter van de kuur gebruikt?** |
| --- |

| ❑ | Nee |
| --- | --- |
| ❑ | Gedeeltelijk |
| ❑ | Ja |
| ❑ | Weet ik niet |

| **36. Heeft u de laatste antibioticumkuur afgemaakt?** |
| --- |

| Dus alle tabletten, pillen of drank opgemaakt. Het kan zijn dat u wel iets heeft overgehouden (in het geval van drank bijvoorbeeld), maar dat u wel de voorgeschreven antibioticumkuur heeft afgemaakt volgens voorschrift. |
| --- |

| ❑ | Nee |
| --- | --- |
| ❑ | Ja |
| ❑ | Nog bezig met de kuur |

|  |
| --- |

| **37. Wat was de belangrijkste reden waarom u de kuur niet hebt afgemaakt?** |
| --- |

| (meerdere antwoorden mogelijk) |
| --- |

| ❑ | Mijn klachten waren verdwenen |
| --- | --- |
| ❑ | Ik had last van bijwerkingen |
| ❑ | Ik heb een ander antibioticum voorgeschreven gekregen |
| ❑ | Ik ben gestopt op advies van mijn arts |
| ❑ | Ik ben gestopt op advies van mijn partner/vriend(in)/familielid |
| ❑ | Anders, namelijk ___________ |

|  |
| --- |

| **38. Wat was de naam van het een-na-laatst gebruikte antibioticum?** |
| --- |

| ❑ | penicilline (Penidural®) |
| --- | --- |
| ❑ | feneticilline (Broxil®) |
| ❑ | flucloxacilline (Floxapen®) |
| ❑ | amoxicilline |
| ❑ | amoxicilline-clavulaanzuur (Augmentin®) |
| ❑ | nitrofurantoïne (Furadantine®, Furabid®) |
| ❑ | doxycycline |
| ❑ | azitromycine (Zithromax®) |
| ❑ | ciprofloxacine (Ciproxin®) |
| ❑ | ceftriaxon |
| ❑ | Anders, namelijk ___________ |
| ❑ | Weet ik niet |

| **39. Wat was de dosering van het een-na-laatste antibioticum (per inname keer) in mg?** |
| --- |

| ❑ | 50 mg |
| --- | --- |
| ❑ | 100 mg |
| ❑ | 200 mg |
| ❑ | 250 mg |
| ❑ | 300 mg |
| ❑ | 400 mg |
| ❑ | 500 mg |
| ❑ | 600 mg |
| ❑ | Anders, namelijk ___________ |
| ❑ | Weet ik niet |

| **40. Hoeveel dagen heeft u de een-na-laatste antibioticumkuur, die u voorgeschreven kreeg, gebruikt?** |
| --- |

| ❑ | 1 |
| --- | --- |
| ❑ | 2 |
| ❑ | 3 |
| ❑ | 4 |
| ❑ | 5 |
| ❑ | 6 |
| ❑ | 7 |
| ❑ | 8 |
| ❑ | 9 |
| ❑ | 10 |
| ❑ | 11 |
| ❑ | 12 |
| ❑ | 13 |
| ❑ | 14 |
| ❑ | Meer dan 14 |
| ❑ | Weet ik niet |

| **41. Hoeveel keer per dag nam u uw een-na-laatste antibioticumkuur?** |
| --- |

| ❑ | 1 |
| --- | --- |
| ❑ | 2 |
| ❑ | 3 |
| ❑ | 4 |
| ❑ | Meer dan 4 keer |
| ❑ | Weet ik niet |

|  |
| --- |

| **42. Welke andere antibiotica heeft u, buiten de al eerder genoemde, verder nog genomen de afgelopen 12 maanden?** |
| --- |

| (meerder antwoorden mogelijk) |
| --- |

| ❑ | penicilline (Penidural®) |
| --- | --- |
| ❑ | feneticilline (Broxil®) |
| ❑ | flucloxacilline (Floxapen®) |
| ❑ | amoxicilline |
| ❑ | amoxicilline-clavulaanzuur (Augmentin®) |
| ❑ | nitrofurantoïne (Furadantine®, Furabid®) |
| ❑ | doxycycline azitromycine (Zithromax®) |
| ❑ | ciprofloxacine (Ciproxin®) |
| ❑ | ceftriaxon |
| ❑ | Anders, namelijk ___________ |
| ❑ | Weet ik niet |

|  |
| --- |

| **43. Heeft u ooit de restjes van een antibioticumkuur bewaard?** |
| --- |

| ❑ | Nee |
| --- | --- |
| ❑ | Ja |
| ❑ | Weet ik niet |

|  |
| --- |

| **44. Gebruikt u weleens restjes van antibioticakuren voor de behandeling van klachten bij uzelf, uw gezinsleden of huisdier?** |
| --- |

| Indien ja, meerder antwoorden mogelijk |
| --- |

| ❑ | Nee |
| --- | --- |
| ❑ | Ja, bij mijzelf |
| ❑ | Ja, bij een of meerdere van mijn gezinsleden |
| ❑ | Ja, bij een of meerdere van mijn huisdieren |

|  |
| --- |

| **GEZONDHEIDSKLACHTEN** |
| --- |

| Hieronder wordt gevraagd of u in de afgelopen 4 weken gezondheidsklachten heeft gehad en, zo ja, om welke klachten het gaat. |
| --- |

| **45. Kruis de klachten aan waarvan u de afgelopen 4 weken last heeft gehad** |
| --- |

| (meerdere antwoorden mogelijk) |
| --- |

| ❑ | Overgeven of braken |
| --- | --- |
| ❑ | Misselijkheid |
| ❑ | Buikpijn/buikkrampen |
| ❑ | Slijm in de ontlasting |
| ❑ | Bloed in de ontlasting |
| ❑ | Lichte (ontkleurde) ontlasting (stopverf) |
| ❑ | Verstopping |
| ❑ | Diarree (minstens 3x per dag) |
| ❑ | Koorts (minstens 38 graden) |
| ❑ | Slap of grieperig gevoel |
| ❑ | Verkoudheid |
| ❑ | Kortademigheid of benauwdheid |
| ❑ | Langdurig hoesten |
| ❑ | Keelpijn |
| ❑ | Misselijkheid |
| ❑ | Hoofdpijn |
| ❑ | Spierpijn (niet door sporten) |
| ❑ | Pijn in de borst |
| ❑ | Langdurige niesbuien (niet door hooikoorts) |
| ❑ | Oorpijn |
| ❑ | Loopoor |
| ❑ | Ontstoken oog |
| ❑ | Anders, namelijk ___________ |
| ❑ | Geen van bovenstaande klachten |

|  |
| --- |

| **46. Hoeveel dagen (ongeveer) heeft u last gehad van deze klachten in de afgelopen 4 weken?** |
| --- |

| ❑ | 1 dag |
| --- | --- |
| ❑ | 2 dagen |
| ❑ | 3 dagen |
| ❑ | 4 dagen |
| ❑ | 5 dagen |
| ❑ | 6 dagen |
| ❑ | 7 dagen |
| ❑ | 8 dagen |
| ❑ | 9 dagen |
| ❑ | 10 dagen |
| ❑ | 11 dagen |
| ❑ | 12 dagen |
| ❑ | 13 dagen |
| ❑ | 14 dagen |
| ❑ | Meer dan 14 dagen |
| ❑ | Weet ik niet |

| **47. Heeft u de afgelopen 4 weken contact gehad met een arts vanwege uw klachten?** |
| --- |

| ❑ | Nee |
| --- | --- |
| ❑ | Ja, 1 keer |
| ❑ | Ja, 2 keer |
| ❑ | Ja, 3 keer |
| ❑ | Ja, 4 keer |
| ❑ | Ja, 5 keer |
| ❑ | Ja, vaker dan 5 keer |

| **48. Heeft u de afgelopen 4 weken een of meer dagdelen in het ziekenhuis gelegen als gevolg van uw klachten?** |
| --- |

| ❑ | Nee |
| --- | --- |
| ❑ | Ja, 1 keer |
| ❑ | Ja, 2 keer |
| ❑ | Ja, 3 keer |
| ❑ | Ja, 4 keer |
| ❑ | Ja, 5 keer |
| ❑ | Ja, vaker dan 5 keer |

| **49. Heeft u de afgelopen 4 weken een medicijn ingenomen om uw klachten te verlichten/te verhelpen?** |
| --- |

| ❑ | Nee |
| --- | --- |
| ❑ | Ja |

|  |
| --- |

| **50. Kon u dit medicijn alleen op recept van een arts krijgen?** |
| --- |

| ❑ | Nee |
| --- | --- |
| ❑ | Ja |
| ❑ | Weet ik niet |

| **51. Wat was de naam van dit medicijn?** |
| --- |

| ❑ | Paracetamol |
| --- | --- |
| ❑ | Ibuprofen |
| ❑ | Anders namelijk ___________ |
| ❑ | Weet ik niet |

| **52. Hoeveel dagen Heeft u dit medicijn ingenomen?** |
| --- |

| ❑ | 1 dag |
| --- | --- |
| ❑ | 2 dagen |
| ❑ | 3 dagen |
| ❑ | 4 dagen |
| ❑ | 5 dagen |
| ❑ | 6 dagen |
| ❑ | 7 dagen |
| ❑ | 8 dagen |
| ❑ | 9 dagen |
| ❑ | 10 dagen |
| ❑ | 11 dagen |
| ❑ | 12 dagen |
| ❑ | 13 dagen |
| ❑ | 14 dagen |
| ❑ | Meer dan 14 dagen |
| ❑ | Weet ik niet |

| **53. Heeft u de afgelopen 4 weken nog een ander medicijn ingenomen om uw klachten te verlichten/te verhelpen?** |
| --- |

| ❑ | Nee |
| --- | --- |
| ❑ | Ja |
| ❑ | Weet ik niet |

|  |
| --- |

| **54. Kon u dit medicijn alleen op recept van een arts krijgen?** |
| --- |

| ❑ | Nee |
| --- | --- |
| ❑ | Ja |
| ❑ | Weet ik niet |

| **55. Wat was de naam van dit medicijn?** |
| --- |

| ❑ | Paracetamol |
| --- | --- |
| ❑ | Ibuprofen |
| ❑ | Anders, namelijk ___________ |
| ❑ | Weet ik niet |

| **56. Hoeveel dagen heeft u dit medicijn ingenomen?** |
| --- |

| ❑ | 1 dag |
| --- | --- |
| ❑ | 2 dagen |
| ❑ | 3 dagen |
| ❑ | 4 dagen |
| ❑ | 5 dagen |
| ❑ | 6 dagen |
| ❑ | 7 dagen |
| ❑ | 8 dagen |
| ❑ | 9 dagen |
| ❑ | 10 dagen |
| ❑ | 11 dagen |
| ❑ | 12 dagen |
| ❑ | 13 dagen |
| ❑ | 14 dagen |
| ❑ | Meer dan 14 dagen |
| ❑ | Weet ik niet |

|  |
| --- |

| **57. Is er de afgelopen 4 weken lichaamsmateriaal bij u afgenomen in verband met uw klachten?** |
| --- |

| (bijvoorbeeld bloed, urine of ontlasting) |
| --- |

| ❑ | Nee |
| --- | --- |
| ❑ | Ja |

|  |
| --- |

| **58. Welk lichaamsmateriaal is afgenomen?** |
| --- |

| (meerdere antwoorden mogelijk) |
| --- |

| ❑ | Bloed |
| --- | --- |
| ❑ | Urine |
| ❑ | Ontlasting |
| ❑ | Anders namelijk ___________ |

|  |
| --- |

| **59. Wat was de uitslag van het ontlastingsonderzoek?** |
| --- |

| (meerdere antwoorden mogelijk) |
| --- |

| ❑ | Niets gevonden |
| --- | --- |
| ❑ | Campylobacter |
| ❑ | Salmonella |
| ❑ | Shigella |
| ❑ | Yersinia |
| ❑ | Vibrio |
| ❑ | Clostridium difficile |
| ❑ | Aeromonas |
| ❑ | Escherichia coli |
| ❑ | Rotavirus |
| ❑ | Norovirus |
| ❑ | Adenovirus typen 40/41 |
| ❑ | Astrovirus |
| ❑ | Entamoeba histolytica |
| ❑ | Giardia lamblia |
| ❑ | Cryptosporidien |
| ❑ | Cyclospora |
| ❑ | Anders, namelijk: ___________ |
| ❑ | Weet ik niet |

|  |
| --- |

| **60. Heeft u in het buitenland overnacht in de 7 dagen voor het begin van uw klachten?** |
| --- |

| ❑ | Nee |
| --- | --- |
| ❑ | Ja |

|  |
| --- |

| **61. In welk land heeft u overnacht in de 7 dagen voor het begin van uw klachten?** |
| --- |
|  |

| **62. Bent u in de afgelopen 4 weken in aanraking geweest met iemand met maag- en/of darmklachten?** |
| --- |

| ❑ | Nee |
| --- | --- |
| ❑ | Ja |
| ❑ | Weet ik niet |

|  |
| --- |

| **OPLEIDING EN WERK** |
| --- |

| De volgende vragen gaan over opleiding en werk. Mocht u deze vragenlijst invullen voor uw (pleeg)kind jonger dan 18 jaar, dan graag uw eigen gegevens gebruiken. |
| --- |

| **63. Wat is uw hoogst voltooide opleiding?** |
| --- |

| ❑ | Basisschool (lager onderwijs, speciaal onderwijs) |
| --- | --- |
| ❑ | Lager beroepsonderwijs (bijvoorbeeld LTS, LHNO, LEAO, huishoudschool) |
| ❑ | Middelbaar algemeen onderwijs (bijvoorbeeld VMBO, ULO, MULO, MAVO) |
| ❑ | Middelbaar beroepsonderwijs (bijvoorbeeld MTS, MEAO, MHNO). |
| ❑ | Voortgezet algemeen onderwijs (bijvoorbeeld HBS, MMS, HAVO, VWO, gymnasium) |
| ❑ | Hoger beroepsonderwijs (bijvoorbeeld HTS, HEAO, HHNO) |
| ❑ | Wetenschappelijk onderwijs |
| ❑ | Geen opleiding afgemaakt |
| ❑ | Deze vraag beantwoord ik liever niet |

| **64. Heeft u een betaalde baan?** |
| --- |

| ❑ | Nee |
| --- | --- |
| ❑ | Ja |

|  |
| --- |

| **65. Hoeveel uur per week doet u dit werk?** |
| --- |

| (bij flexibele werktijden het gemiddelde nemen) |
| --- |

| ongeveer | _________________________ |
| --- | --- |

|  |
| --- |

| **66. In welke bedrijfstak bent u werkzaam?** |
| --- |

| (meerder antwoorden mogelijk) |
| --- |

| ❑ | Gezondheids- of welzijnszorg |
| --- | --- |
| ❑ | Onderwijs |
| ❑ | Kinderopvang |
| ❑ | Dierverzorging/dierenwelzijn |
| ❑ | Horeca |
| ❑ | Bosbouw/bosbeheer |
| ❑ | Landbouw |
| ❑ | Veehouderij |
| ❑ | Vleesverwerking (incl. slachthuis) |
| ❑ | Transport van dieren/landbouwproducten |
| ❑ | Zuivelindustrie |
| ❑ | Wol-,vilt- of leerverwerking |
| ❑ | Bouw/wegenbouw |
| ❑ | (detail)handel |
| ❑ | Zakelijke dienstverlening |
| ❑ | Overheid en politiek |
| ❑ | Industrie en energie |
| ❑ | Afvalverwerking |
| ❑ | Waterbeheer |
| ❑ | Plaagdier- en ongediertebestrijding |
| ❑ | Anders |

| **67. Heeft u een beroep waarbij u met groepen kinderen in aanraking komt?** |
| --- |

| (Bijvoorbeeld leraar, crèche-medewerker, consultatieverpleegkundige, etc.) |
| --- |

| ❑ | Nee |
| --- | --- |
| ❑ | Ja |

|  |
| --- |

| **68. Heeft u tijdens uw werkzaamheden in de gezondheids- of welzijnszorg direct contact met de patiënten/cliënten/bewoners?** |
| --- |

| (bijvoorbeeld helpende, verzorgende, verpleegkundige, arts) |
| --- |

| ❑ | Nee |
| --- | --- |
| ❑ | Ja |

|  |
| --- |

| **69. Wat is uw functie?** |
| --- |

| ❑ | Helpende |
| --- | --- |
| ❑ | Verzorgende |
| ❑ | Verpleegkundige (niveau 4 of 5) |
| ❑ | Verpleegkundig specialist |
| ❑ | Fysiotherapeut |
| ❑ | Arts |
| ❑ | Doktersassistent(e) |
| ❑ | Ergotherapeut |
| ❑ | Diëtist |
| ❑ | Anders, namelijk ___________ |

| **70. In wat voor een instelling werkt u?** |
| --- |

| (meerdere antwoorden mogelijk) |
| --- |

| ❑ | Ziekenhuis |
| --- | --- |
| ❑ | Verpleeghuis |
| ❑ | Verzorgingstehuis |
| ❑ | Revalidatiecentrum |
| ❑ | Huisartsenpraktijk |
| ❑ | Thuiszorg |
| ❑ | Consultatiebureau |
| ❑ | GGD |
| ❑ | GGZ |
| ❑ | Anders namelijk ___________ |

|  |
| --- |

| **71. Heeft u een beroep waarbij u met (landbouw)huisdieren in aanraking komt?** |
| --- |

| (bijvoorbeeld veehouder, dierenarts, hondentrimmer, etc.) |
| --- |

| ❑ | Nee |
| --- | --- |
| ❑ | Ja |

|  |
| --- |

| **72. Met welke dieren komt u beroepsmatig in aanraking?** |
| --- |

| (meerder antwoorden mogelijk) |
| --- |

| ❑ | Hond(en) |
| --- | --- |
| ❑ | Kat(ten) |
| ❑ | Konijn(en) |
| ❑ | Muis/muizen (als huisdier) |
| ❑ | Rat(ten) (als huisdier) |
| ❑ | Cavia(‘s) |
| ❑ | Hamster(s) |
| ❑ | Koe(ien) |
| ❑ | Schaap/schapen |
| ❑ | Kip(pen) |
| ❑ | Vogels (als huisdier) |
| ❑ | Varken(s) |
| ❑ | Geit(en) |
| ❑ | Hert(en) |
| ❑ | Paard(en)/pony(‘s) |
| ❑ | Ezel(s) |
| ❑ | Reptielen |
| ❑ | Vissen |
| ❑ | Insecten |
| ❑ | Anders, namelijk ___________ |

|  |
| --- |

| **73. Heeft een beroep waarbij u voedsel voor anderen bereidt?** |
| --- |

| ❑ | Nee |
| --- | --- |
| ❑ | Ja |

| **74. Heeft u een beroep waarbij u met rauw vlees in aanraking komt?** |
| --- |

| (bijvoorbeeld slager, vleesverwerkingsindustrie, etc) |
| --- |

| ❑ | Nee |
| --- | --- |
| ❑ | Ja |

|  |
| --- |

| **75. Met welk(e) rauwe dierproduct(en) komt u in aanraking?** |
| --- |

| ❑ | Rundvlees |
| --- | --- |
| ❑ | Schapenvlees |
| ❑ | Kippenvlees |
| ❑ | Varkensvlees |
| ❑ | Geitenvlees |
| ❑ | Konijnenvlees |
| ❑ | Paardenvlees |
| ❑ | Anders, namelijk ___________ |

|  |
| --- |

| **CONTACT MET DIEREN EN VLEES** |
| --- |

| De volgende vragen gaan over contact met (landbouw)huisdieren en vlees.    LET OP! Deze vragen zijn gericht aan de persoon met geboortedatum [Geboortedatum] |
| --- |

| **76. Houdt u (landbouw)huisdieren in of rondom uw huis?** |
| --- |

| (meerdere antwoorden mogelijk) |
| --- |

| ❑ | Nee |
| --- | --- |
| ❑ | Hond(en) |
| ❑ | Kat(ten) |
| ❑ | Konijn(en) |
| ❑ | Muis/muizen (als huisdier) |
| ❑ | Rat(ten) (als huisdier) |
| ❑ | Cavia(‘s) |
| ❑ | Hamster(s) |
| ❑ | Koe(ien) |
| ❑ | Schaap/schapen |
| ❑ | Kip(pen) |
| ❑ | Ander pluimvee (als landbouwdieren) |
| ❑ | Vogels (als huisdier) |
| ❑ | Varken(s) |
| ❑ | Geit(en) |
| ❑ | Hert(en) |
| ❑ | Paard(en)/pony(‘s) |
| ❑ | Ezel(s) |
| ❑ | Reptielen |
| ❑ | Vissen |
| ❑ | Insecten |
| ❑ | Anders, namelijk: ___________ |

| **77. Heeft u in de afgelopen vier weken direct contact gehad met (landbouw)huisdieren?** |
| --- |

| (met direct contact wordt hier bedoeld dat u uw huisdier aanraakt, bijvoorbeeld door te aaien of te knuffelen met uw huisdier) |
| --- |

| ❑ | Nee |
| --- | --- |
| ❑ | Ja |
| ❑ | Weet ik niet |

|  |
| --- |

| **78. Met welke (landbouw)huisdieren heeft u de afgelopen 4 weken contact gehad?** |
| --- |

| (meerdere antwoorden mogelijk) |
| --- |

| ❑ | Hond(en) |
| --- | --- |
| ❑ | Kat(ten) |
| ❑ | Konijn(en) |
| ❑ | Muis/muizen (als huisdier) |
| ❑ | Rat(ten) (als huisdier) |
| ❑ | Cavia(‘s) |
| ❑ | Hamster(s) |
| ❑ | Koe(ien) |
| ❑ | Schaap/schapen |
| ❑ | Kip(pen) |
| ❑ | Ander pluimvee (als landbouwdieren) |
| ❑ | Vogels (als huisdier) |
| ❑ | Varken(s) |
| ❑ | Geit(en) |
| ❑ | Hert(en) |
| ❑ | Paard(en)/pony(‘s) |
| ❑ | Ezel(s) |
| ❑ | Reptielen |
| ❑ | Vissen |
| ❑ | Insecten |
| ❑ | Anders, namelijk: ___________ |

|  |
| --- |

| **79. Heeft u in de afgelopen vier weken in uw omgeving ratten- en/of muizenuitwerpselen gezien?** |
| --- |

| (meerdere antwoorden mogelijk) |
| --- |

| ❑ | Nee |
| --- | --- |
| ❑ | Ja, binnenshuis |
| ❑ | Ja, om/bij het huis |
| ❑ | Op een andere locatie, namelijk ___________ |

| **80. Bent u in de afgelopen vier weken in contact geweest met onderstaande dieren/(dier)producten?** |
| --- |

| wanneer u contact heeft gehad met meerdere diersoorten, graag alle diersoorten vermelden |
| --- |

| ❑ | Mest/uitwerpselen van landbouwhuisdieren (bijvoorbeeld tijdens schoonmaken van hokken/stallen of het bemesten van uw tuin) |
| --- | --- |
| ❑ | Compost |
| ❑ | Hooi/stro |
| ❑ | Dode dieren, namelijk: (diersoort) ___________ |
| ❑ | Dieren die pas (dood of levend geboren) jongen hebben gekregen, namelijk (diersoort) ___________ |
| ❑ | Placentamateriaal, namelijk: (diersoort) ___________ |
| ❑ | Onbehandelde wol, namelijk: (diersoort) ___________ |
| ❑ | Dierenvacht of –huid van eend ood dier, namelijk: (diersoort) ___________ |
| ❑ | Geen van bovenstaande |

|  |
| --- |

| **VOEDING** |
| --- |

| De volgende vragen gaan over de eetgewoonten van de aangeschreven persoon met geboortedatum [Geboortedatum] |
| --- |

| **81. Welk eetpatroon is op u/de geadresseerde van toepassing?** |
| --- |

| (gelatine is een bindmiddel verkregen uit dierlijke producten. In niet-vegetarische kaas is stremsel, verkregen uit kalvermaag verwerkt) |
| --- |

| ❑ | U eet nooit producten waar een dier voor gedood moest worden en u eet ook geen andere dierlijke producten (zoals zuivel, eieren en honing) |
| --- | --- |
| ❑ | U eet nooit producten waar dieren voor gedood zijn, zoals vlees, vis, kaas en gelatine |
| ❑ | U eet nooit vlees en vis, maar (soms) wel kaas en/of producten waar gelatine in verwerkt is |
| ❑ | U eet nooit vlees, maar (soms) wel vis, kaas en/of producten waar gelatine in verwerkt is |
| ❑ | U eet wel vlees en/of vis, maar eet één of meerdere dagen per week geen vlees en/of vis |
| ❑ | U eet dagelijks vlees |
| ❑ | Geen van bovenstaande |

|  |
| --- |

| **82. Hoe vaak eet u de volgende vleesproducten?** |
| --- |

|  | Nooit | Minder vaak dan 1 keer per week | 1-3 Keer per week | 4-6 Keer per week | 7-8 Keer per week | Vaker dan 8 keer per week |
| --- | --- | --- | --- | --- | --- | --- |
| 1. Kip | ❑ | ❑ | ❑ | ❑ | ❑ | ❑ |
| 2. Varkensvlees | ❑ | ❑ | ❑ | ❑ | ❑ | ❑ |
| 3. Rundvlees | ❑ | ❑ | ❑ | ❑ | ❑ | ❑ |
| 4. (Half) rauwe vleesproducten (bijvoorbeeld biefstuk, gehakt, filet americain, rosbief, rauwe ham, ossenworst) | ❑ | ❑ | ❑ | ❑ | ❑ | ❑ |

|  |
| --- |

| **83. Hoe vaak eet u de volgende visproducten?** |
| --- |

|  | Nooit | Minder vaak dan 1 keer per week | 1-3 Keer per week | 4-6 Keer per week | 7-8 Keer per week | Vaker dan 8 keer per week |
| --- | --- | --- | --- | --- | --- | --- |
| 1. Vis | ❑ | ❑ | ❑ | ❑ | ❑ | ❑ |
| 2. Schelpdierproducten (zoals mossels, oesters) | ❑ | ❑ | ❑ | ❑ | ❑ | ❑ |
| 3. Schaaldieren (zoals krab, kreeft, garnalen) | ❑ | ❑ | ❑ | ❑ | ❑ | ❑ |

|  |
| --- |

| **84. Hoe vaak eet/drinkt u de volgende producten?** |
| --- |

|  | Nooit | Minder vaak dan 1 keer per week | 1-3 Keer per week | 4-6 Keer per week | 7-8 Keer per week | Vaker dan 8 keer per week |
| --- | --- | --- | --- | --- | --- | --- |
| 1. Doorverhitte eieren | ❑ | ❑ | ❑ | ❑ | ❑ | ❑ |
| 2. Rauwe eieren | ❑ | ❑ | ❑ | ❑ | ❑ | ❑ |
| 3. Zuivelproducten | ❑ | ❑ | ❑ | ❑ | ❑ | ❑ |

|  |
| --- |

| **85. Hoe vaak eet u de volgende producten?** |
| --- |

|  | Nooit | Minder vaak dan 1 keer per week | 1-3 Keer per week | 4-6 Keer per week | 7-8 Keer per week | Vaker dan 8 keer per week. |
| --- | --- | --- | --- | --- | --- | --- |
| 1. Groente(producten)? | ❑ | ❑ | ❑ | ❑ | ❑ | ❑ |
| 2. Fruit(producten)? | ❑ | ❑ | ❑ | ❑ | ❑ | ❑ |
| 3. Rauwkost (bijvoorbeeld sla, komkommer, tomaat, wortel, tuinkruiden)? | ❑ | ❑ | ❑ | ❑ | ❑ | ❑ |

| **86. Heeft u in de afgelopen week vleesproducten gegeten die rauw of niet goed doorbakken waren?** |
| --- |

| Rauw voedsel betekent dat het helemaal niet doorbakken is. Niet goed doorbakken vlees betekent dat het nog steeds roze/rood van binnen is en/of er bloederige/rode sappen uitlekken.  (meerdere antwoorden mogelijk) |
| --- |

| ❑ | Nee |
| --- | --- |
| ❑ | Ja, rundvlees |
| ❑ | Ja, varkensvlees |
| ❑ | Ja, kippenvlees |
| ❑ | Ja, kalkoenvlees |
| ❑ | Ja, orgaanvlees |
| ❑ | Ja, wildvlees |
| ❑ | Ja, anders namelijk ___________ |

| **87. Als u vlees eet, is dit meestal** |
| --- |

| ❑ | Regulier vlees van de slager, supermarkt of toko |
| --- | --- |
| ❑ | Biologisch vlees van de slager, supermarkt of toko |
| ❑ | Vlees uit een winkel direct verbonden aan een veehouderij |
| ❑ | Weet ik niet |

| **88. Als u groenten eet, is dit meestal:** |
| --- |

| ❑ | Reguliere groenten van de groentewinkel, supermarkt of toko |
| --- | --- |
| ❑ | Biologische groenten van de biologische winkel, supermarkt of toko |
| ❑ | Groenten uit een winkel direct verbonden aan een boerderij |
| ❑ | Groenten uit een eigen moestuin |
| ❑ | Weet ik niet |

| **89. Hoeveel opscheplepels eet u ongeveer wanneer u groente(producten) eet?** |
| --- |

| ❑ | Minder dan 1 opscheplepel (minder dan 50 gram) |
| --- | --- |
| ❑ | 1-2 Opscheplepels (ongeveer 51-100 gram) |
| ❑ | 3-4 Opscheplepels (ongeveer 101-200 gram) |
| ❑ | Meer dan 4 opscheplepels (meer dan 200 gram) |
| ❑ | Weet ik niet |

|  |
| --- |

| **90. Hoe vaak eet u op jaarbasis een diner in een restaurant of andere commerciële eetgelegenheden?** |
| --- |

| (afhaalmaaltijden niet meegerekend) |
| --- |

| ❑ | Nooit |
| --- | --- |
| ❑ | 1 tot 3 keer |
| ❑ | 4 tot 12 keer |
| ❑ | 13 tot 20 keer |
| ❑ | Meer dan 20 keer |

| **91. Bij welke supermarkt wordt er binnen uw huishouden het vaakst boodschappen gedaan?** |
| --- |

| ❑ | Albert Heijn |
| --- | --- |
| ❑ | Aldi |
| ❑ | C1000 |
| ❑ | Coop |
| ❑ | Jumbo |
| ❑ | Lidl |
| ❑ | Plus |
| ❑ | Nettorama |
| ❑ | Hoogvliet |
| ❑ | Troefmarkt |
| ❑ | Attent |
| ❑ | DeKaMarkt |
| ❑ | Dirk van de Broek |
| ❑ | Bas van de Heijden |
| ❑ | Emté |
| ❑ | Anders, namelijk ___________ |
| ❑ | Niet van toepassing |

|  |
| --- |

| **92. Welke producten worden er gekocht in de supermarkt die u hiervoor heeft ingevuld?** |
| --- |

| (meerdere antwoorden mogelijk) |
| --- |

| ❑ | Vlees |
| --- | --- |
| ❑ | Vis |
| ❑ | Schaal/schelpdieren |
| ❑ | Groente |
| ❑ | Fruit |
| ❑ | Geen van bovenstaande |

| **93. Ligt deze supermarkt waar u het vaakst komt in uw eigen woonplaat?** |
| --- |

| ❑ | Nee, het ligt in ___________ |
| --- | --- |
| ❑ | Ja |

|  |
| --- |

| **94. Bij welke supermarkt wordt er het één-na-vaakst boodschappen gedaan in uw huishouden?** |
| --- |

| ❑ | Albert Heijn |
| --- | --- |
| ❑ | Aldi |
| ❑ | C1000 |
| ❑ | Coop |
| ❑ | Jumbo |
| ❑ | Lidl |
| ❑ | Plus |
| ❑ | Nettorama |
| ❑ | Hoogvliet |
| ❑ | Troefmarkt |
| ❑ | Attent |
| ❑ | DeKaMarkt |
| ❑ | Dirk van de Broek |
| ❑ | Bas van de Heijden |
| ❑ | Emté |
| ❑ | Anders, namelijk ___________ |
| ❑ | Geen van bovenstaande |

|  |
| --- |

| **95. Welke producten worden er gekocht in de supermarkt die u hiervoor heeft ingevuld?** |
| --- |

| (meerdere antwoorden mogelijk) |
| --- |

| ❑ | Vlees |
| --- | --- |
| ❑ | Vis |
| ❑ | Schaal/schelpdieren |
| ❑ | Groente |
| ❑ | Fruit |
| ❑ | Geen van bovenstaande |

| **96. Is deze supermarkt in uw eigen woonplaats gelegen?** |
| --- |

| ❑ | Nee, het ligt in: ___________ |
| --- | --- |
| ❑ | Ja |

|  |
| --- |

| **HYGIËNE** |
| --- |

| De volgende vragen gaan over keuken- en schoonmaakgewoonten van zowel uzelf/de geadresseerde als het gehele huishouden. |
| --- |

| **97. Hoeveel tijd zit er meestal tussen het kopen van gekoelde en/of diepgevroren etenswaren en het opbergen ervan in de koelkast en vriezer?** |
| --- |

| ❑ | Minder dan een kwartier |
| --- | --- |
| ❑ | Een kwartier tot een half uur |
| ❑ | Een half uur tot een uur |
| ❑ | 1 uur tot 2 uur |
| ❑ | Meer dan 2 uur |
| ❑ | Weet ik niet |

| **98. Hoe vaak wordt de koelkast gereinigd?** |
| --- |

| Het kan zijn dat u dit zelf doet of dat een ander dit doet. Vul hieronder in wat van toepassing is. |
| --- |

| ❑ | Wekelijks |
| --- | --- |
| ❑ | Maandelijks |
| ❑ | Elke 3 maanden |
| ❑ | Minder vaak dan elke 3 maanden |

| **99. Wast u (of degene die kookt in uw huishouden) uw handen:** |
| --- |

| Opmerking: indien u zelf geen vlees eet, kan het nog steeds zo zijn dat u vlees voor anderen klaarmaakt. |
| --- |

|  | Altijd | Meestal | Soms | Zelden | Nooit |
| --- | --- | --- | --- | --- | --- |
| 1. Voordat u met het bereiden van voedsel begint | ❑ | ❑ | ❑ | ❑ | ❑ |
| 2. Na het snijden of bereiden van rauw vlees, voordat bijvoorbeeld rauwe groenten bereid worden | ❑ | ❑ | ❑ | ❑ | ❑ |

|  |
| --- |

| **100. Van welk materiaal is de meest gebruikte snijplank gemaakt?** |
| --- |

| Ook hier kan het zijn dat u dit zelf doet of dat iemand anders dit voor u doet. Vul in wat van toepassing is in uw huishouden. |
| --- |

| ❑ | Hout |
| --- | --- |
| ❑ | Plastic/kunststof |
| ❑ | Glas |
| ❑ | Aardewerk |
| ❑ | Bamboe |
| ❑ | Anders, namelijk ___________ |

| **101. Wordt dezelfde snijplank gebruikt voor het snijden van rauw vlees en voor ander voedsel tijdens de bereiding van dezelfde maaltijd?** |
| --- |

| (Indien u zelf geen vlees eet, kan het nog steeds zo zijn dat u vlees voor anderen klaarmaakt). |
| --- |

| ❑ | Nee |
| --- | --- |
| ❑ | Ja, ik gebruik dezelfde snijplank |
| ❑ | Ja, ik draai de plank om of was hem tussendoor af |
| ❑ | Niet van toepassing, ik gebruik geen snijplank |

| **102. Wordt het mes gebruikt voor rauw vlees ook voor het snijden van ander voedsel gebruikt?** |
| --- |

| (indien u zelf geen vlees eet, kan het nog steeds zo zijn dat u vlees voor anderen klaarmaakt) |
| --- |

| ❑ | Nee |
| --- | --- |
| ❑ | Ja, en ik maak het mes tussendoor schoon |
| ❑ | Ja, en ik maak het mes tussendoor niet schoon |
| ❑ | Niet van toepassing |

|  |
| --- |

| **103. Hoe vaak wordt er gemiddeld:** |
| --- |

| (met schoon wordt hier wasmachineschoon bedoeld) |
| --- |

|  | Elke dag | Om de dag | Meer dan 1 keer per week | 1 keer per 1 à 2 weken | 1 keer per 3 à 4 weken | Minder dan 1 keer per maand | Niet van toepassing |
| --- | --- | --- | --- | --- | --- | --- | --- |
| 1. Een schoon handdoekje gepakt in uw huishouden voor in de keuken? (handdoekje met als doel om handen af te drogen) | ❑ | ❑ | ❑ | ❑ | ❑ | ❑ | ❑ |
| 2. Een schoon vaatdoekje of schuursponsje gebruikt in uw huishouden? | ❑ | ❑ | ❑ | ❑ | ❑ | ❑ | ❑ |
| 3. Een schoon handdoekje gepakt binnen uw huishouden voor in het toilet? | ❑ | ❑ | ❑ | ❑ | ❑ | ❑ | ❑ |

| **104. Wast u uw handen na toiletbezoek?** |
| --- |

| ❑ | Altijd |
| --- | --- |
| ❑ | Meestal |
| ❑ | Soms |
| ❑ | Zelden |
| ❑ | Nooit |
| ❑ | Niet van toepassing (door bijvoorbeeld het dragen van een luier) |

| **105. Hoe vaak wordt het toilet in uw huis gereinigd?** |
| --- |

| ❑ | Elke dag |
| --- | --- |
| ❑ | Om de dag |
| ❑ | Meer dan 1 keer per week |
| ❑ | 1 keer per 1 a 2 weken |
| ❑ | 1 keer per 3 a 4 weken |
| ❑ | Minder dan 1 keer per maand |

|  |
| --- |

| **ACTIVITEITEN** |
| --- |

| De volgende vragen gaan over of u en, zo ja, welke activiteiten u/uw gezin de afgelopen tijd heeft ondernomen. |
| --- |

| **106. Bent u in de afgelopen 12 maanden in het buitenland geweest?** |
| --- |

| (1 overnachting of meer) |
| --- |

| ❑ | Nee |
| --- | --- |
| ❑ | Ja |

|  |
| --- |

| **107. Hoe vaak bent u in het buitenland geweest in de afgelopen 12 maanden?** |
| --- |

| ❑ | 1 keer |
| --- | --- |
| ❑ | 2 keer |
| ❑ | 3 keer |
| ❑ | 4 keer |
| ❑ | 5 keer |
| ❑ | 6 keer |
| ❑ | Vaker dan 6 keer |

| **108. Naar welk land bent u het kortst geleden geweest in de afgelopen 12 maanden (1e land)?** |
| --- |
| **109. Wat was het doel van deze reis?** |

| (doel behorend bij het hierboven ingevulde land) |
| --- |

| ❑ | Vakantiereis |
| --- | --- |
| ❑ | Werkgerelateerde reis |
| ❑ | Studiegerelateerde reis |
| ❑ | Familie-/vriendenbezoek |
| ❑ | Anders, namelijk ___________ |

| **110. Hoe lang verbleef u daar?** |
| --- |

| ❑ | Minder dan een week |
| --- | --- |
| ❑ | 1-2 weken |
| ❑ | 2-3 weken |
| ❑ | 4-5 weken |
| ❑ | 5-8 weken |
| ❑ | 9-10 weken |
| ❑ | 10-12 weken |
| ❑ | Meer dan 12 weken |

| **111. In welke maand kwam u terug van uw reis?** |
| --- |

| ❑ | Januari |
| --- | --- |
| ❑ | Februari |
| ❑ | Maart |
| ❑ | April |
| ❑ | Mei |
| ❑ | Juni |
| ❑ | Juli |
| ❑ | Augustus |
| ❑ | September |
| ❑ | Oktober |
| ❑ | November |
| ❑ | December |

|  |
| --- |

| **112. Vink hieronder aan naar welk land u nog meer geweest bent in de afgelopen 12 maanden (2e land):** |
| --- |

| **113. Wat was het doel van deze reis?** |
| --- |

| (doel behorend bij het hierboven ingevulde land) |
| --- |

| ❑ | Vakantiereis |
| --- | --- |
| ❑ | Werkgerelateerde reis |
| ❑ | Studiegerelateerde reis |
| ❑ | Anders, namelijk ___________ |

| **114. Hoe lang verbleef u daar?** |
| --- |

| ❑ | Minder dan een week |
| --- | --- |
| ❑ | 1-2 weken |
| ❑ | 2-3 weken |
| ❑ | 4-5 weken |
| ❑ | 5-8 weken |
| ❑ | 9-10 weken |
| ❑ | 10-12 weken |
| ❑ | Meer dan 12 weken |

| **115. In welke maand kwam u terug van uw reis?** |
| --- |

| ❑ | Januari |
| --- | --- |
| ❑ | Februari |
| ❑ | Maart |
| ❑ | April |
| ❑ | Mei |
| ❑ | Juni |
| ❑ | Juli |
| ❑ | Augustus |
| ❑ | September |
| ❑ | Oktober |
| ❑ | November |
| ❑ | December |

|  |
| --- |

| **116. Naar welke landen bent u nog meer geweest, maar heeft u hierboven niet meer in kunnen vullen?** |
| --- |
| **117. Hoe vaak heeft u in de afgelopen 12 maanden de onderstaande activiteiten ondernomen?** |

|  | 0 keer | 1 keer | 2-3 keer | 4-12 keer | vaker dan 12 keer |
| --- | --- | --- | --- | --- | --- |
| Op een camping of kampeerterrein of in de buitenlucht overnacht (meerdere aaneengesloten nachten telt als één keer) | ❑ | ❑ | ❑ | ❑ | ❑ |
| Gezwommen in open zoetwater (bijvoorbeeld meer, rivier, maar niet in een zwembad) | ❑ | ❑ | ❑ | ❑ | ❑ |
| Gezwommen in open zoutwater (zee) | ❑ | ❑ | ❑ | ❑ | ❑ |
| In het bos geweest | ❑ | ❑ | ❑ | ❑ | ❑ |
| Kinderboerderij bezocht | ❑ | ❑ | ❑ | ❑ | ❑ |
| Speeltuin bezocht (kan een speelpark zijn of een buurtspeeltuintje) | ❑ | ❑ | ❑ | ❑ | ❑ |

| **118. Hoe vaak heeft u de afgelopen 4 weken aan onderstaande activiteiten deelgenomen?** |
| --- |

| Als u een activiteit heeft ondernomen, graag invullen (ongeveer) hoe vaak dit was in de afgelopen 4 weken. Heeft u geen van onderstaande activiteiten ondernomen, kunt u 0 invullen.  Let op: dit gaat over de afgelopen 4 weken |
| --- |

|  | 0 keer | 1 keer | 2 tot 3 keer | 4 tot 12 keer | vaker dan 12 keer |
| --- | --- | --- | --- | --- | --- |
| Een tuincentrum bezocht | ❑ | ❑ | ❑ | ❑ | ❑ |
| In de tuin gewerkt | ❑ | ❑ | ❑ | ❑ | ❑ |
| In het bos geweest | ❑ | ❑ | ❑ | ❑ | ❑ |
| In een vakantiehuisje of recreatiepark geweest | ❑ | ❑ | ❑ | ❑ | ❑ |
| Op een camping of kampeerterrein of in de buitenlucht overnacht (meerdere aaneengesloten nachten telt als één keer) | ❑ | ❑ | ❑ | ❑ | ❑ |
| Op een golfterrein geweest | ❑ | ❑ | ❑ | ❑ | ❑ |
| Gezwommen in open zout water (zee) | ❑ | ❑ | ❑ | ❑ | ❑ |
| Gezwommen in open zoet water (bijvoorbeeld meer, rivier, maar niet in een zwembad) | ❑ | ❑ | ❑ | ❑ | ❑ |
| Een sauna, bubbelbad of zwembad bezocht | ❑ | ❑ | ❑ | ❑ | ❑ |
| Ergens ander dan thuis gedoucht (bijvoorbeeld op werk, sportlocatie, bij familie) | ❑ | ❑ | ❑ | ❑ | ❑ |
| Een autowasstraat bezocht | ❑ | ❑ | ❑ | ❑ | ❑ |
| Met een hoge drukspuit, plantenspuit of tuinslang gewerkt | ❑ | ❑ | ❑ | ❑ | ❑ |
| Aan de verwarming of waterleidingen geklust / laten klussen? | ❑ | ❑ | ❑ | ❑ | ❑ |
| Kinderboerderij bezocht | ❑ | ❑ | ❑ | ❑ | ❑ |
| Speeltuin bezocht (kan een speelpark zijn of een buurtspeeltuintje) | ❑ | ❑ | ❑ | ❑ | ❑ |

|  |
| --- |

| **LABORATORIUMONDERZOEK** |
| --- |

| In ontlasting (poep) komen veel bacteriën voor. Daarom is ontlasting geschikt om te onderzoeken in het laboratorium op de aanwezigheid van (resistente) bacteriën en ziekteverwekkers. Iedereen kan resistente bacteriën bij zich dragen.  Om een goed beeld te krijgen van de (resistente) bacteriën en ziekteverwekkers die u bij u draagt, willen wij u vriendelijk vragen mee te doen aan het ontlastingsonderzoek. Hierbij zal u worden benaderd om voor uzelf (of de geadresseerde persoon), een kleine hoeveelheid ontlasting (poep) te verzamelen.  Wanneer u besluit mee te doen, ontvangt u een pakketje met alle benodigdheden inclusief een instructie en verzend envelop. U maakt dus zelf geen kosten en u kunt aangeven of u de uitslag teruggekoppeld wilt krijgen. U zou ons erg helpen door onderstaande vragen in te vullen.  Bij voorbaat hartelijk dank! |
| --- |

| **119. Bent u bereid binnenkort mee te werken aan een ontlastingsonderzoek (poeponderzoek) naar resistente bacteriën en ziekteverwekkers bij uzelf (de geadresseerde)?** |
| --- |

| Indien u ja invult, krijgt u na ongeveer een maand meer informatie over de verdere procedure. |
| --- |

| ❑ | Nee |
| --- | --- |
| ❑ | Ja |

|  |
| --- |

| **TOESTEMMINGSVERKLARING** |
| --- |

| Om het onderzoek uit te mogen voeren, is het belangrijk dat onderstaande vragen ingevuld worden. |
| --- |

| **120. In welke leeftijdscategorie valt u/de geadresseerde?** |
| --- |

| ❑ | 0-12 |
| --- | --- |
| ❑ | 13-17 |
| ❑ | 18 en ouder |

| **121. Ik wil op de hoogte gehouden worden van de onderzoeksresultaten en rapportage van de algemene onderzoeksresultaten (dus niet uw persoonlijke resultaten).** |
| --- |

| ❑ | Nee |
| --- | --- |
| ❑ | Ja |

| **122. Ik ga ermee akkoord dat de adresgegevens uit deze verklaring gebruikt worden om, eventueel benaderd te worden voor een vervolgonderzoek.** |
| --- |

| ❑ | Nee |
| --- | --- |
| ❑ | Ja |

|  |
| --- |

| **123. Ouder/verzorger 1: gaat u ook akkoord met bovenstaande punten, zoals ingevuld door uw kind?** |
| --- |

| ❑ | Nee |
| --- | --- |
| ❑ | Ja |

| **124. Ouder/verzorger 2: gaat u ook akkoord met bovenstaande punten, zoals ingevuld door uw kind?** |
| --- |

| ❑ | Nee |
| --- | --- |
| ❑ | Ja |
| ❑ | Niet van toepassing |

|  |
| --- |

| **125. Ouder/verzorger 2: Gaat u ook akkoord met bovenstaande punten, zoals ingevuld door de andere ouder/verzorger van uw kind (indien van toepassing)?** |
| --- |

| ❑ | Nee |
| --- | --- |
| ❑ | Ja |
| ❑ | Niet van toepassing |

|  |
| --- |

| **126. Wat is uw emailadres?** |
| --- |

| Dit is met name belangrijk:  - wanneer u mee wilt doen aan het ontlastingsonderzoek  - we u nog mogen benaderen wanneer er aanvullende vragen zijn  - u op de hoogte gehouden wilt worden van de algemene onderzoeksresultaten |
| --- |

| ______________________________ |
| --- |
| ______________________________ |
| ______________________________ |
| ______________________________ |

| **127. Herhaling emailadres** |
| --- |

| ______________________________ |
| --- |
| ______________________________ |
| ______________________________ |
| ______________________________ |

| **128. Heeft u nog opmerkingen?** |
| --- |

| ______________________________ |
| --- |
| ______________________________ |
| ______________________________ |
| ______________________________ |
