# Supplementary material for: Incidence and economic burden of community-acquired gastroenteritis in the Netherlands: Does having children in the household make a difference?
Source: PLoS One. 2019 May 23;14(5):e0217347. doi: 10.1371/journal.pone.0217347 (PMC6532970; doi:10.1371/journal.pone.0217347)
Supplement: S2 Questionnaire — (DOC) [file pone.0217347.s002.doc]

Population study on antibiotic resistance

**Population study on antibiotic resistance**

Welcome!

Participation in this study is entirely voluntary. If you participate you have the right to revoke your consent at any time without having to give a reason for it.

If you have any questions and / or problems, you can contact the researchers of the UMCU and the RIVM by telephone or (preferably) by e-mail. You will find the contact details of the researchers in the letter you received.

Your data will remain anonymous and will not be provided to third parties.

Thank you very much for your cooperation!

**These are the topics that we will ask questions about. Depending on your answers, some parts take less time to complete than others.**

* General questions

* Family and household

* Health

* Antibiotics use

* Health complaints

* Education and work

* Contact with animals and meat

* Food

* Hygiene

* Travel and other activities

* Laboratory research

* Pet questions

At the end of the questionnaire we ask for your permission to use your answers for our research. Depending on the age of the invited person, we also need permission from the parent (s) / caregiver (s).

GENERAL QUESTIONS

**1. Is this questionnaire completed by the invited person? (with date of birth [Date of birth])**

❑ No.

❑ Yes

**2. What is your relationship with the invited person?**

Do you want to fill in the questions so that it is correct for the invited person?

❑ Parent / caretaker

❑ Brother / sister

❑ Son / daughter

❑ Partner

❑ Other ___________

**3. Is the date of birth of the invited person:**

[Date of birth]

❑ This is correct

❑ This is not correct. It should be: ___________

**4. What is your country of birth?**

(If your country of birth is not in the list, choose the 'Other' option at the bottom)

**5. My country of birth is:**

______________________________

**FAMILY AND HOUSEHOLD**

The following questions are about your household (the household of the person with [Date of birth] as date of birth)

**6. How many persons (living at home) does your household have?**

(including yourself / addressee)

❑ 1

❑ 2

❑ 3

❑ 4

❑ 5

❑ 6

❑ 7

❑ 8

❑ 9

❑ 10

❑ More than 10

**7. What is your living situation?**

❑ I live in a single-family house (house, semi-detached house, detached house)

❑ I live in an apartment

❑ I live in a student residence

❑ I live in a nursing or care home

❑ Other, namely: ___________

❑ I live on a farm

**8. Are there children (living at home) in your household?**

❑ No.

❑ Yes

**9. How many children (living at home) does your household have?**

❑ 1

❑ 2

❑ 3

❑ 4

❑ 5

❑ 6

❑ 7

❑ 8

❑ More than 8

**10. How old are the children in your household?**

Multiple answers possible

❑ 0 to 12 months

❑ Between 1 and 2 years

❑ 3 to 12 years

❑ 13 to 18 years

❑ Older than 18 years

**11. Are there children in your household who go to a day care center?**

(multiple answers possible)

❑ No.

❑ Yes, the invited person

❑ Yes, a child from the household

**12. Are there children in your household who go to a playgroup/kindergarten?**

(multiple answers possible)

❑ No.

❑ Yes, the invited person

❑ Yes, a child from the household

**13. Are there children in your household who go to a guest parent?**

(multiple answers possible)

❑ No.

❑ Yes, the invited person

❑ Yes, a child from the household

**14. How many children in your household wear diapers?**

❑ 0

❑ 1

❑ 2

❑ 3

❑ More than 3

**15. Does someone smoke in your household?**

❑ No.

❑ Yes, outdoors

❑ Yes, indoors

❑ I do not know

**16. Which of the options below applies to you?**

(multiple answer options possible)

❑ My home and / or work address is near a forest

❑ My home and / or work address is located near a city park / city forest

❑ My home and / or work address is next to a pasture

❑ My home and / or work address is located near a cropland

❑ My home and / or work address is near water (sea, lake, river, stream, canal, etc.)

❑ None of the above

**HEALTH**

Below are questions about your health (the person with date of birth [Date of birth])

**17. Have you been admitted to a healthcare institution in the past 12 months? (1 night or more)**

(Multiple answers possible)

❑ No.

❑ Yes, in a hospital

❑ Yes, in a nursing home

❑ Yes, in a care home

❑ Yes, in a rehabilitation center

❑ Yes, otherwise ___________

**18. How many times have you been admitted in the past 12 months?**

(1 night or more)

❑ 1

❑ 2

❑ 3

❑ 4

❑ 5

❑ 6

❑ 7

❑ 8

❑ 9

❑ 10

❑ More than 10 times

**19. Was / were the admission (s) abroad?**

❑ This was in the Netherlands

❑ This was abroad

**20. In which country (s) were you admitted?**

(multiple answers possible)

**21. What type of department were you admitted to?**

(multiple answers possible)

❑ On a short stay (short-stay department)

❑ In a general nursing ward (eg oncology, urology, surgery, internal, nephrology, lung, rheumatism, stomach, intestine and liver, cardiology department, etc.)

❑ In an intensive care unit (IC) or cardiac monitoring unit (CCU)

❑ Other, namely ___________

**22. Was one of the other family members (living at home) admitted in the last 12 months (1 night or more)?**

(Multiple answers possible)

❑ No.

❑ Yes, in a hospital

❑ Yes, in a nursing home

❑ Yes, in a care home

❑ Yes, in a rehabilitation center

❑ Yes, otherwise ___________

**23. Which long-term health complaints and / or illnesses apply to you (with date of birth [Date of birth])**

(multiple answers possible)

❑ Disease of the gastrointestinal tract

❑ Food allergy

❑ Diabetes (diabetes)

❑ Absence of the spleen

❑ Liver disease

❑ Kidney disease

❑ Heart and / or vascular diseases

❑ Cancer

❑ Immune disorder

❑ Rheumatism

❑ Lung disease

❑ Other, namely ___________

❑ No complaints

**24. Have you had medication on doctor's prescription in the past 6 months?**

(Multiple answers possible)

❑ No.

❑ Yes, medication due to an infection (eg antibiotics)

❑ Yes, antacids (such as omeprazole, pantoprazole, lansoprazole, rabeprazole, esomeprazole)

❑ Yes, cholesterol-lowering drugs (such as simvastatin, pravastatin, atorvastatin)

❑ Yes, laxatives (eg colophort, endofalk, movicolon, molaxole)

❑ Yes, antihypertensive drugs (such as betaxolol, valsartan, amlodipine, etc.)

❑ Yes, ADHD medication (like ritalin)

❑ Yes, antidepressants (such as venlafaxine, lithium, MAO inhibitors, etc.)

❑ Yes, sedatives and tranquillizers (such as diazepam, oxazepam, temazepam, etc.)

❑ Yes, antidiabetics (such as metformin, glimepiride, levemir, novorapid, etc.)

❑ Yes, chemotherapy

❑ Yes, contraceptive pill (for women only)

❑ Yes, otherwise ___________

**ANTIBIOTIC USAGE**

Antibiotics are prescribed for treatment of certain infections. The doctor prescribes an antibiotic in a certain amount and for a certain number of days. This is called an antibiotic treatment.

Examples of antibiotics include penicillin (Penidural®), pheneticillin (Broxil®), flucloxacillin (Floxapen®), amoxicillin, amoxicillin-clavulanic acid (Augmentin®), nitrofurantoin (Furadantine®, Furabid®), doxycycline, azithromycin (Zithromax®) and ciprofloxacin (Ciproxin®). It is possible that you have used or know antibiotics that are not listed here. There are many more antibiotics on the market.

PAY ATTENTION! The following questions are about the person invited in the letter (with date of birth [Date of birth])

**25. When did you use antibiotics for the last time?**

❑ Never

❑ The last time was less than a month ago

❑ The last time was 1 to 3 months ago

❑ The last time was 4 to 6 months ago

❑ The last time was 6 months to a year ago

❑ The last time was more than a year ago

**26. How often have you used an antibiotic treatment in the last 12 months?**

(If a treatment is extended, you can consider the total course as 1 time)

❑ 1 time

❑ 2 times

❑ 3 times

❑ 4 times

❑ 5 times

❑ More than 5 times

**27. What was the name of the last used antibiotic?**

❑ penicillin (Penidural®)

❑ pheneticillin (Broxil®)

❑ flucloxacillin (Floxapen®)

❑ amoxicillin

❑ amoxicillin clavulanic acid (Augmentin®)

❑ nitrofurantoin (Furadantine®, Furabid®)

❑ doxycycline

❑ azithromycin (Zithromax®)

❑ ciprofloxacin (Ciproxin®)

❑ ceftriaxone

❑ Other, namely ___________

❑ I do not know

**28. What was the dose of this antibiotic (per intake) in mg?**

❑ 50 mg

❑ 100 mg

❑ 200 mg

❑ 250 mg

❑ 300 mg

❑ 400 mg

❑ 500 mg

❑ 600 mg

❑ Other, namely ___________

❑ I do not know

**29. How many days did you use the last prescribed antibiotic treatment?**

❑ 1

❑ 2

❑ 3

❑ 4

❑ 5

❑ 6

❑ 7

❑ 8

❑ 9

❑ 10

❑ 11

❑ 12

❑ 13

❑ 14

❑ More than 14

❑ I do not know

**30. How many times a day did you take your last antibiotic treatment?**

❑ 1

❑ 2

❑ 3

❑ 4

❑ More than 4 times

❑ I do not know

**31. For which infection have you been prescribed the last antibiotic treatment?**

❑ Urinary tract infection (bladder infection)

❑ Pneumonia

❑ Laryngitis

❑ Gastrointestinal infection

❑ Other, namely ___________

**32. How many times have you had a bladder infection during the past 6 months?**

❑ 1

❑ 2

❑ 3

❑ 4

❑ More than 4

**33. How did you obtain the last antibiotic treatment?**

❑ By prescription from the GP

❑ By prescription from the attending physician at the hospital

❑ By prescription of dentist

❑ By prescription via the traveler’s consultation

❑ By prescription of relative / family member

❑ Via internet without a prescription

❑ Abroad without a prescription

❑ From someone else's leftover

❑ Other, namely ___________

**34. With the last antibiotics treatment, did someone tell you how to use the antibiotic?**

(multiple answers possible)

❑ No.

❑ Yes, the doctor or assistant of the general practitioner

❑ Yes, the attending doctor or assistant in the hospital

❑ Yes, the dentist or dental assistant

❑ Yes, the pharmacist or pharmacist's assistant

❑ Yes, someone else, namely ___________

**35. Did you use the last antibiotic treatment according to the package leaflet?**

❑ No.

❑ Partially

❑ Yes

❑ I do not know

**36. Have you completed the last antibiotic treatment?**

So all tablets, pills or drinks were consumed. It may be that you have something left over (in the case of a drink for example), but that you have completed the prescribed antibiotic treatment according to prescription.

❑ No.

❑ Yes

❑ Treatment still ongoing

**37. What was the main reason why you did not complete the course?**

(multiple answers possible)

❑ My complaints were gone

❑ I suffered from side effects

❑ I have been prescribed another antibiotic

❑ I stopped on the advice of my doctor

❑ I stopped on the advice of my partner / friend / family member

❑ Other, namely ___________

**38. What was the name of the second-to-last used antibiotic?**

❑ penicillin (Penidural®)

❑ pheneticillin (Broxil®)

❑ flucloxacillin (Floxapen®)

❑ amoxicillin

❑ amoxicillin clavulanic acid (Augmentin®)

❑ nitrofurantoin (Furadantine®, Furabid®)

❑ doxycycline

❑ azithromycin (Zithromax®)

❑ ciprofloxacin (Ciproxin®)

❑ ceftriaxone

❑ Other, namely ___________

❑ I do not know

**39. What was the dose of the second-last antibiotic (per intake) in mg?**

❑ 50 mg

❑ 100 mg

❑ 200 mg

❑ 250 mg

❑ 300 mg

❑ 400 mg

❑ 500 mg

❑ 600 mg

❑ Other, namely ___________

❑ I do not know

**40. How many days did you use the second-to-last antibiotic treatment prescribed to you?**

❑ 1

❑ 2

❑ 3

❑ 4

❑ 5

❑ 6

❑ 7

❑ 8

❑ 9

❑ 10

❑ 11

❑ 12

❑ 13

❑ 14

❑ More than 14

❑ I do not know

**41. How many times a day did you take your second-to-last antibiotic treatment?**

❑ 1

❑ 2

❑ 3

❑ 4

❑ More than 4 times

❑ I do not know

**42. What other antibiotics have you taken in addition to the aforementioned, in the past 12 months?**

(more answers possible)

❑ penicillin (Penidural®)

❑ pheneticillin (Broxil®)

❑ flucloxacillin (Floxapen®)

❑ amoxicillin

❑ amoxicillin clavulanic acid (Augmentin®)

❑ nitrofurantoin (Furadantine®, Furabid®)

❑ doxycycline azithromycin (Zithromax®)

❑ ciprofloxacin (Ciproxin®)

❑ ceftriaxone

❑ Other, namely ___________

❑ I do not know

**43. Have you ever preserved the remains of an antibiotic treatment?**

❑ No.

❑ Yes

❑ I do not know

**44. Do you ever use leftovers of antibiotics for the treatment of complaints in yourself, your family members or pets?**

If yes, multiple answers possible

❑ No.

❑ Yes, for myself

❑ Yes, for one or more of my family members

❑ Yes, for one or more of my pets

HEALTH COMPLAINTS

Below you will be asked whether you have had health complaints in the past 4 weeks and, if so, what complaints were involved.

**45. Please tick the complaints that you have suffered from in the last 4 weeks**

(multiple answers possible)

❑ Vomiting

❑ Nausea

❑ Abdominal pain / abdominal cramps

❑ Mucus in the stool

❑ Blood in the stool

❑ Light (decolored) stool

❑ Constipation

❑ Diarrhea (at least 3 times a day)

❑ Fever (at least 38 degrees)

❑ Sluggish or flu-like feeling

❑ Cold

❑ Shortness of breath

❑ Long-term coughing

❑ Sore throat

❑ Nausea

❑ Headache

❑ Muscle pain (not through sports)

❑ Pain in the chest

❑ Prolonged sneezing (not by hay fever)

❑ Earache

❑ Running ear

❑ Inflamed eye

❑ Other, namely ___________

❑ None of the above complaints

**46. ​​How many days (approximately) did you suffer from these complaints in the past 4 weeks?**

❑ 1 day

❑ 2 days

❑ 3 days

❑ 4 days

❑ 5 days

❑ 6 days

❑ 7 days

❑ 8 days

❑ 9 days

❑ 10 days

❑ 11 days

❑ 12 days

❑ 13 days

❑ 14 days

❑ More than 14 days

❑ I do not know

**47. Did you have contact with a doctor in the past 4 weeks due to your complaints?**

❑ No.

❑ Yes, once

❑ Yes, twice

❑ Yes, 3 times

❑ Yes, 4 times

❑ Yes, 5 times

❑ Yes, more than 5 times

**48. Did you have one or more hospital admissions during the past 4 weeks as a result of your complaints?**

❑ No.

❑ Yes, once

❑ Yes, twice

❑ Yes, 3 times

❑ Yes, 4 times

❑ Yes, 5 times

❑ Yes, more than 5 times

**49. Have you taken medication in the past 4 weeks to relieve your symptoms or as a remedy?**

❑ No.

❑ Yes

**50. Could you only receive this medication on prescription from a doctor?**

❑ No.

❑ Yes

❑ I do not know

**51. What was the name of this medicine?**

❑ Paracetamol

❑ Ibuprofen

❑ Otherwise ___________

❑ I do not know

**52. How many days did you take this medicine?**

❑ 1 day

❑ 2 days

❑ 3 days

❑ 4 days

❑ 5 days

❑ 6 days

❑ 7 days

❑ 8 days

❑ 9 days

❑ 10 days

❑ 11 days

❑ 12 days

❑ 13 days

❑ 14 days

❑ More than 14 days

❑ I do not know

**53. Have you taken any other medication in the past 4 weeks to relieve your symptoms or as a remedy?**

❑ No.

❑ Yes

❑ I do not know

**54. Could you only receive this medication on prescription from a doctor?**

❑ No.

❑ Yes

❑ I do not know

**55. What was the name of this medicine?**

❑ Paracetamol

❑ Ibuprofen

❑ Other, namely ___________

❑ I do not know

**56. How many days did you take this medicine?**

❑ 1 day

❑ 2 days

❑ 3 days

❑ 4 days

❑ 5 days

❑ 6 days

❑ 7 days

❑ 8 days

❑ 9 days

❑ 10 days

❑ 11 days

❑ 12 days

❑ 13 days

❑ 14 days

❑ More than 14 days

❑ I do not know

**57. Have any samples been taken in the past 4 weeks due to your complaints?**

(eg blood, urine or stool)

❑ No.

❑ Yes

**58. What samples were taken?**

(multiple answers possible)

❑ Blood

❑ Urine

❑ Feces

❑ Otherwise ___________

**59. What was the outcome of the stool examination?**

(multiple answers possible)

❑ Nothing found

❑ Campylobacter

❑ Salmonella

❑ Shigella

❑ Yersinia

❑ Vibrio

❑ Clostridium difficile

❑ Aeromonas

❑ Escherichia coli

❑ Rotavirus

❑ Norovirus

❑ Adenovirus types 40/41

❑ Astrovirus

❑ Entamoeba histolytica

❑ Giardia lamblia

❑ Cryptosporidien

❑ Cyclospora

❑ Other, namely: ___________

❑ I do not know

**60. Have you stayed overnight abroad in the 7 days before the start of your complaints?**

❑ No.

❑ Yes

**61. In which country did you stay overnight in the 7 days before the start of your complaints?**

**62. Have you been in contact with someone with stomach and / or intestinal complaints in the past 4 weeks?**

❑ No.

❑ Yes

❑ I do not know

**EDUCATION AND WORK**

The following questions are about education and work. If you fill in this questionnaire for your (foster) child under the age of 18, please fill in your own data.

**63. What is your highest level of education?**

❑ Primary school (primary education, special education)

❑ Lower vocational education (for example LTS, LHNO, LEAO, housekeeping school)

❑ Lower secondary education (eg VMBO, ULO, MULO, MAVO)

❑ Intermediate vocational education (eg MTS, MEAO, MHNO).

❑ Intermediate or higher secondary education (eg HBS, MMS, HAVO, VWO, gymnasium)

❑ Higher vocational education (eg HTS, HEAO, HHNO)

❑ University education

❑ No training completed

❑ I would rather not answer this question

**64. Do you have a paid job?**

❑ No.

❑ Yes

**65. How many hours per week do you work?**

(average for flexible working hours)

approximately _________________________

**66. In which sector do you work?**

(multiple answers possible)

❑ Health or well-being

❑ Education

❑ Childcare

❑ Animal care / animal welfare

❑ Catering

❑ Forestry / forest management

❑ Agriculture

❑ Livestock farming

❑ Meat processing (including slaughterhouse)

❑ Transport of animals / agricultural products

❑ Dairy industry

❑ Wool, felt or leather processing

❑ Construction / road construction

❑ Trade

❑ Business services

❑ Government and politics

❑ Industry and energy

❑ Waste processing

❑ Water management

❑ Pest and pest control

❑ Otherwise

**67. Do you have a profession in which you come into contact with groups of children?**

(For example, teacher, crèche employee, consultation nurse, etc.)

❑ No.

❑ Yes

**68. Do you have direct contact with the patients / clients / residents during your work in health or social care?**

(eg, nurse, doctor)

❑ No.

❑ Yes

**69. What is your profession?**

❑ Assisting role

❑ Caring

❑ Nurse (level 4 or 5)

❑ Nursing specialist

❑ Physical therapist

❑ Doctor

❑ Doctor's assistant (e)

❑ Ergo therapist

❑ Dietitian

❑ Other, namely ___________

**70. In what kind of institution do you work?**

(multiple answers possible)

❑ Hospital

❑ Nursing home

❑ Care home

❑ Rehabilitation center

❑ GP practice

❑ Home care

❑ Consultancy

❑ GGD

❑ GGZ

❑ Otherwise ___________

**71. Do you have a profession in which you come into contact with farm animals or pets?**

(eg cattle farmer, veterinarian, dog groomer, etc.)

❑ No.

❑ Yes

**72. With which animals do you have occupational contact?**

(multiple answers possible)

❑ Dog (s)

❑ Cat (s)

❑ Rabbit (s)

❑ Mouse / mice (as a pet)

❑ Rat (s) (as a pet)

❑ Guinea pig (s)

❑ Hamster (s)

❑ Cow/cattle

❑ Sheep

❑ Chicken (s)

❑ Birds (as pets)

❑ Pig (s)

❑ Goat (s)

❑ Deer

❑ Horse (s) / pony (s)

❑ Donkey (s)

❑ Reptiles

❑ Fish

❑ Insects

❑ Other, namely ___________

**73. Do you have an occupation where you prepare food for others?**

❑ No.

❑ Yes

**74. Do you have a profession where you come into contact with raw meat?**

(for example butcher, meat processing industry, etc)

❑ No.

❑ Yes

**75. With which raw animal product (s) do you come into contact?**

❑ Beef

❑ Mutton

❑ Chicken meat

❑ Pork

❑ Goat meat

❑ Rabbit meat

❑ Horse meat

❑ Other, namely ___________

**CONTACT WITH ANIMALS AND MEAT**

The following questions are about contact with pets, farms animals, and meat.

PAY ATTENTION! These questions are addressed to the person with date of birth [Date of birth]

**76. Do you keep farm animals or pets in or around your home?**

(multiple answers possible)

❑ No.

❑ Dog (s)

❑ Cat (s)

❑ Rabbit (s)

❑ Mouse / mice (as a pet)

❑ Rat (s) (as a pet)

❑ Guinea pig (s)

❑ Hamster (s)

❑ Cow/cattle

❑ Sheep

❑ Chicken (s)

❑ Other poultry (as farm animals)

❑ Birds (as pets)

❑ Pig (s)

❑ Goat (s)

❑ Deer

❑ Horse (s) / pony (s)

❑ Donkey (s)

❑ Reptiles

❑ Fish

❑ Insects

❑ Other, namely: ___________

**77. Have you had direct contact with farm animals or pets in the past four weeks?**

(direct contact means touching your pet, for example by petting or cuddling with your pet)

❑ No.

❑ Yes

❑ I do not know

**78. Which farm animals or pets did you have contact with in the past 4 weeks?**

(multiple answers possible)

❑ Dog (s)

❑ Cat (s)

❑ Rabbit (s)

❑ Mouse / mice (as a pet)

❑ Rat (s) (as a pet)

❑ Guinea pig (s)

❑ Hamster (s)

❑ Cow/cattle

❑ Sheep

❑ Chicken (s)

❑ Other poultry (as farm animals)

❑ Birds (as pets)

❑ Pig (s)

❑ Goat (s)

❑ Deer

❑ Horse (s) / pony (s)

❑ Donkey (s)

❑ Reptiles

❑ Fish

❑ Insects

❑ Other, namely: ___________

**79. Have you seen rat and / or mouse excreta in your area in the past four weeks?**

(multiple answers possible)

❑ No.

❑ Yes, indoors

❑ Yes, around / near the house

❑ At another location, namely ___________

**80. Have you been in contact with the following animals / (animal) products in the past four weeks?**

if you have had contact with several animal species, please mention all animal species

❑ Manure / excrement of farm animals (eg during cleaning of pens / stalls or fertilizing your garden)

❑ Compost

❑ Hay / straw

❑ Dead animals, namely: (animal species) ___________

❑ Animals that recently gave birth (dead or alive), namely (animal species) ___________

❑ Placenta material, namely: (animal species) ___________

❑ Untreated wool, namely: (animal species) ___________

❑ Animal fur or skin of a dead animal, namely: (animal species) ___________

❑ None of the above

**DIET**

The following questions are about the eating habits of the person addressed with the date of birth [Date of birth]

**81. Which eating habits apply to you / the addressee?**

(gelatin is a binder product obtained from animal products. In non-vegetarian cheese, rennet obtained from calf stomach is processed)

❑ You never eat products for which an animal has to be killed and you do not eat any other animal products (such as dairy, eggs and honey)

❑ You never eat products for which an animal has to be killed, such as meat, fish, cheese and gelatin

❑ You never eat meat and fish, but (sometimes) cheese and / or products that contain gelatin

❑ You never eat meat, but (sometimes) fish, cheese and / or products in which gelatin is processed

❑ You eat meat and / or fish, but do not eat meat and / or fish one or more days a week

❑ You eat meat daily

❑ None of the above

**82. How often do you eat the following meat products?**

[Never] [Less often than once a week] [1-3 Times a week] [4-6 Times a week] [7-8 Times a week] [More than 8 times a week]

1. Chicken ❑ ❑ ❑ ❑ ❑ ❑

2. Pork ❑ ❑ ❑ ❑ ❑ ❑

3. Beef ❑ ❑ ❑ ❑ ❑ ❑

4. (Semi) raw meat products (for example steak, minced meat, filet americain, roast beef, raw ham, beef sausage) ❑ ❑ ❑ ❑ ❑ ❑

**83. How often do you eat the following fish products?**

[Never] [Less often than once a week] [1-3 Times a week] [4-6 Times a week] [7-8 Times a week] [More than 8 times a week]

1. Fish ❑ ❑ ❑ ❑ ❑ ❑

2. Shellfish (such as mussels, oysters) ❑ ❑ ❑ ❑ ❑ ❑

3. Crustaceans (such as crab, lobster, shrimp) ❑ ❑ ❑ ❑ ❑ ❑

**84. How often do you eat / drink the following products?**

[Never] [Less often than once a week] [1-3 Times a week] [4-6 Times a week] [7-8 Times a week] [More than 8 times a week]

1. Heated eggs ❑ ❑ ❑ ❑ ❑ ❑

2. Raw eggs ❑ ❑ ❑ ❑ ❑ ❑

3. Dairy products ❑ ❑ ❑ ❑ ❑ ❑

**85. How often do you eat the following products?**

[Never] [Less often than once a week] [1-3 Times a week] [4-6 Times a week] [7-8 Times a week] [More than 8 times a week]

1. Vegetable (products)? ❑ ❑ ❑ ❑ ❑ ❑

2. Fruit (products)? ❑ ❑ ❑ ❑ ❑ ❑

3. Raw vegetables (eg lettuce, cucumber, tomato, carrot, garden herbs)? ❑ ❑ ❑ ❑ ❑ ❑

**86. Did you eat meat products that were raw or not properly cooked in the past week?**

Raw food means that it is not cooked/baked. Not well cooked meat means that it is still pink / red on the inside and / or bleeding / red juices are leaking.

(multiple answers possible)

❑ No.

❑ Yes, beef

❑ Yes, pork

❑ Yes, chicken meat

❑ Yes, turkey meat

❑ Yes, organ meat

❑ Yes, game meat

❑ Yes, otherwise ___________

**87. If you eat meat, this is usually**

❑ Regular meat from the butcher, supermarket or toko

❑ Organic meat from the butcher, supermarket or toko

❑ Meat from a store directly connected to a livestock farm

❑ I do not know

**88. If you eat vegetables, this is usually:**

❑ Regular vegetables from the greengrocer's, supermarket or toko

❑ Organic vegetables from the organic shop, supermarket or toko

❑ Vegetables from a store directly connected to a farm

❑ Vegetables from a private vegetable garden

❑ I do not know

**89. How much serving spoons do you eat when you eat vegetables (products)?**

❑ Less than 1 serving spoon (less than 50 grams)

❑ 1-2 Serving spoons (about 51-100 grams)

❑ 3-4 Serving spoons (about 101-200 grams)

❑ More than 4 serving spoons (more than 200 grams)

❑ I do not know

**90. How often do you eat dinner in a restaurant or other commercial food establishment annually?**

(not including takeaway meals)

❑ Never

❑ 1 to 3 times

❑ 4 to 12 times

❑ 13 to 20 times

❑ More than 20 times

**91. At which supermarket is your grocery shopping most often done?**

❑ Albert Heijn

❑ Aldi

❑ C1000

❑ Coop

❑ Jumbo

❑ Lidl

❑ Plus

❑ Nettorama

❑ Hoogvliet

❑ Troefmarkt

❑ Attent

❑ DeKaMarkt

❑ Dirk van de Broek

❑ Bas van de Heijden

❑ Emté

❑ Other, namely ___________

❑ Not applicable

**92. Which products are bought in the supermarket filled in above?**

(multiple answers possible)

❑ Meat

❑ Fish

❑ Shellfish

❑ Vegetable

❑ Fruit

❑ None of the above

**93. Is this supermarket located in your own place of residence?**

❑ No, it is in ___________

❑ Yes

**94. At which supermarket do you do your second most shopping in your household?**

❑ Albert Heijn

❑ Aldi

❑ C1000

❑ Coop

❑ Jumbo

❑ Lidl

❑ Plus

❑ Nettorama

❑ Hoogvliet

❑ Troefmarkt

❑ Attent

❑ DeKaMarkt

❑ Dirk van de Broek

❑ Bas van de Heijden

❑ Emté

❑ Other, namely ___________

❑ None of the above

**95. Which products are bought in the supermarket filled in above?**

(multiple answers possible)

❑ Meat

❑ Fish

❑ Shellfish

❑ Vegetable

❑ Fruit

❑ None of the above

**96. Is this supermarket located in your own place of residence?**

❑ No, it is in: ___________

❑ Yes

**HYGIENE**

The following questions are about kitchen and cleaning habits of yourself / the addressee and the entire household.

97. How much time is there usually between buying chilled and / or deep-frozen food and storing it in the fridge and freezer?

❑ Less than a quarter of an hour

❑ A quarter to a half hour

❑ Half an hour to an hour

❑ 1 hour to 2 hours

❑ More than 2 hours

❑ I do not know

**98. How often is the refrigerator cleaned?**

It is possible that you do this yourself or that someone else does it. Enter what is applicable below.

❑ Weekly

❑ Monthly

❑ Every 3 months

❑ Less often than every 3 months

**99. Do you wash (or the person who cooks in your household) your hands:**

Note: if you do not eat meat yourself, you may still be able to prepare meat for others.

[Always] [Usually] [Sometimes] [Rarely] [Never]

1. Before you start cooking food ❑ ❑ ❑ ❑ ❑

2. After cutting or preparing raw meat, before preparing raw vegetables for example ❑ ❑ ❑ ❑ ❑

**100. What material is the most commonly used cutting board made of?**

Again, you may do this yourself or someone else does it for you. Fill in what is applicable in your household.

❑ Wood

❑ Plastic

❑ Glass

❑ Pottery

❑ Bamboo

❑ Other, namely ___________

**101. Is the same cutting board used for cutting raw meat and for other food during the preparation of the same meal?**

(If you do not eat meat yourself, it may still be that you prepare meat for others).

❑ No.

❑ Yes, I use the same cutting board

❑ Yes, I turn the shelf or wash it off in between

❑ Not applicable, I do not use a cutting board

**102. Is the knife that is used for raw meat also used for cutting other foods?**

(if you do not eat meat yourself, it may still be that you prepare meat for others)

❑ No.

❑ Yes, and I clean the knife in between

❑ Yes, and I do not clean the knife in between

❑ Not applicable

**103. How often is there on average:**

(with clean is meant here ‘dishwasher clean’)

[Every day] [Every other day] [More than once a week] [once every 1 to 2 weeks once] [every 3 to 4 weeks] [Less than once a month] [Not applicable]

1. A clean towel for the kitchen in your household? (towel with the aim to dry hands) ❑ ❑ ❑ ❑ ❑ ❑ ❑

2. A clean dishcloth or scourer in your household? ❑ ❑ ❑ ❑ ❑ ❑ ❑

3. A clean toilet towel in your household?

**104. Do you wash your hands after a toilet visit?**

❑ Always

❑ Usually

❑ Sometimes

❑ Rarely

❑ Never

❑ Not applicable (for example, wearing a diaper)

**105. How often is the toilet in your house cleaned?**

❑ Every day

❑ Every other day

❑ More than once a week

❑ 1 time per 1 to 2 weeks

❑ once every 3 to 4 weeks

❑ Less than once a month

**ACTIVITIES**

The following questions are about whether you/your family undertook activities and, if so, what activities.

**106. Have you been abroad in the past 12 months?**

(1 night or more)

❑ No.

❑ Yes

**107. How often have you been abroad in the past 12 months?**

❑ 1 time

❑ 2 times

❑ 3 times

❑ 4 times

❑ 5 times

❑ 6 times

❑ More often than 6 times

**108. Which country were you most recently in the past 12 months (1st country)?**

**109. What was the purpose of this trip?**

❑ Holiday trip

❑ Work-related journey

❑ Study-related journey

❑ Family / friends visit

❑ Other, namely ___________

**110. How long did you stay there?**

❑ Less than a week

❑ 1-2 weeks

❑ 2-3 weeks

❑ 4-5 weeks

❑ 5-8 weeks

❑ 9-10 weeks

❑ 10-12 weeks

❑ More than 12 weeks

**111. In which month did you return from your trip?**

❑ January

❑ February

❑ March

❑ April

❑ May

❑ June

❑ July

❑ August

❑ September

❑ October

❑ November

❑ December

**112. Fill in below to which country you have been in the past 12 months (2nd country):**

**113. What was the purpose of this trip?**

❑ Holiday trip

❑ Work-related journey

❑ Study-related journey

❑ Other, namely ___________

**114. How long did you stay there?**

❑ Less than a week

❑ 1-2 weeks

❑ 2-3 weeks

❑ 4-5 weeks

❑ 5-8 weeks

❑ 9-10 weeks

❑ 10-12 weeks

❑ More than 12 weeks

**115. In which month did you return from your trip?**

❑ January

❑ February

❑ March

❑ April

❑ May

❑ June

❑ July

❑ August

❑ September

❑ October

❑ November

❑ December

**116. Which countries have you been to, but were not able to fill in above?**

**117. How often have you undertaken the following activities in the past 12 months?**

[0 times] [1 time] [2-3 times] [4-12 times] [more than 12 times]

Stayed overnight on a camping, camping site, or in the open air (several consecutive nights count as one time) ❑ ❑ ❑ ❑ ❑

Swimming in surface water (for example lake, river, but not in a swimming pool) ❑ ❑ ❑ ❑ ❑

Swam in salt water (sea) ❑ ❑ ❑ ❑ ❑

Been in the forest ❑ ❑ ❑ ❑ ❑

Visited a petting zoo ❑ ❑ ❑ ❑ ❑

Visited a playground ❑ ❑ ❑ ❑ ❑

**118. How often did you undertake the following activities in the past 4 weeks?**

If you have undertaken an activity, please fill in (approximately) how often this was in the last 4 weeks. If you have not undertaken any of the following activities, you can enter 0.

Note: this is about the last 4 weeks

[0 times] [1 time] [2 to 3 times] [4 to 12 times] [more often than 12 times]

Visited a garden center ❑ ❑ ❑ ❑ ❑

Worked in the garden ❑ ❑ ❑ ❑ ❑

Been in the forest ❑ ❑ ❑ ❑ ❑

Been in a vacation home or recreation park ❑ ❑ ❑ ❑ ❑

On a camping, camping site or in the open air overnight (several consecutive nights count as one time) ❑ ❑ ❑ ❑ ❑

Been on a golf course ❑ ❑ ❑ ❑ ❑

Swam in salt water (sea) ❑ ❑ ❑ ❑ ❑

Swim in surface water (eg lake, river, but not in a swimming pool) ❑ ❑ ❑ ❑ ❑

Visited a sauna, hot tub or swimming pool ❑ ❑ ❑ ❑ ❑

Showed somewhere else than at home (for example at work, sports location, with family) ❑ ❑ ❑ ❑ ❑

Visited a car wash ❑ ❑ ❑ ❑ ❑

Worked with a high pressure cleaner, plant spray or garden hose ❑ ❑ ❑ ❑ ❑

Worked on the heating or water pipes? ❑ ❑ ❑ ❑ ❑

Visited a petting zoo ❑ ❑ ❑ ❑ ❑

Visited a playground visited ❑ ❑ ❑ ❑ ❑

**LABORATORY RESEARCH**

Many bacteria are present in stool (feces). That is why feces is suitable for laboratory testing for the presence of (resistant) bacteria and pathogens. Everyone can carry resistant bacteria.

To gain insight in the (resistant) bacteria and pathogens you carry with you, we kindly ask you to participate in stool examination. You will be asked to collect a small amount of feces (poo) of yourself (or the addressee).

When you decide to participate, you will receive a package with all the necessary items including an instruction and a shipping envelope. You do not incur any costs yourself and you can indicate whether you would like to get the results. You would really help us by filling in the questions below.

Many thanks in advance!

**119. Are you willing to cooperate in feces examination (poop examination) into resistant bacteria and pathogens?**

If you fill in yes, you will receive more information about the further procedure after about a month.

❑ No.

❑ Yes

PERMISSION STATEMENT

In order to be able to carry out the research, it is important that the following questions are answered.

120. In what age category do you / the addressee fall?

❑ 0-12

❑ 13-17

❑ 18 and older

121. I want to be informed of the examination results and reports of the overall research results (not your personal results).

❑ No.

❑ Yes

122. I agree that the address details from this statement are used for follow-up investigation.

❑ No.

❑ Yes

123. Parent / caretaker 1: do you also agree with the above points, as entered by your child?

❑ No.

❑ Yes

124. Parent / caretaker 2: do you also agree with the above points, as entered by your child?

❑ No.

❑ Yes

❑ Not applicable

125. Parent / caregiver 2: Do you also agree with the above points, as entered by your child's other parent / caregiver (if applicable)?

❑ No.

❑ Yes

❑ Not applicable

126. What is your email address?

This is particularly important:

- if you want to participate in the stool examination

- we may contact you if there are additional questions

- you want to be kept informed of the general research results

______________________________

______________________________

______________________________

______________________________

127. Repeat email address

______________________________

______________________________

______________________________

______________________________

128. Do you have any comments?

______________________________

______________________________

______________________________

______________________________
