# Supplementary material for: Incidence and economic burden of community-acquired gastroenteritis in the Netherlands: Does having children in the household make a difference?
Source: PLoS One. 2019 May 23;14(5):e0217347. doi: 10.1371/journal.pone.0217347 (PMC6532970; doi:10.1371/journal.pone.0217347)
Supplement: S1 Table — (DOCX) [file pone.0217347.s003.docx]

**S1 Table.** Unit costs in Euros, 2017

|  | Unit cost (€) | Source |
| --- | --- | --- |
| **Direct Healthcare costs** |  |  |
| General practitioner (per consultation) |  |  |
| Visit | 33.78 | [1] |
| House visit | 51.18 | [1] |
| Phone call | 17.40 | [1] |
| Weighted^a^ | 52.94 | [1, 2] |
| Hospitalization adults (19+ years) per day | 487.23 | [1] |
| Hospitalization children (0-18 years) per day | 641.79 | [1] |
| Medicine prescribed by a doctor | 7.92 | [1, 3] |
| Sample collection + testing | 75.38 | [1, 4] |
| Ambulance emergency transport | 627.46 | [1] |
| **Patient costs** |  |  |
| Medicine over-the-counter | 1.37 | [3] |
| Travel costs per km (car/bus) | 0.19 | [1] |
| Parking fee (car) | 3.07 | [1] |
| **Productivity losses** |  |  |
| Productivity losses from paid work^b^ |  |  |
| 15 – 19 years | 9.60 | [1, 5] |
| 20 – 24 years | 18.65 | [1, 5] |
| 25 – 29 years | 26.47 | [1, 5] |
| 30 – 34 years | 32.64 | [1, 5] |
| 35 – 39 years | 37.58 | [1, 5] |
| 40 – 44 years | 40.72 | [1, 5] |
| 45 – 49 years | 41.90 | [1, 5] |
| 50 – 54 years | 42.32 | [1, 5] |
| 55 – 59 years | 42.55 | [1, 5] |
| 60 – 64 years | 42.00 | [1, 5] |
| 65+ years | 36.41 | [1, 5] |

^a^Weighted cost for general practitioner (GP) visit (90% of cases), GP house visit (10% of cases) and GP telephone consultation (97% of cases).

b Derived based on the average person productivity losses as given by Case Institute Netherlands (in Dutch: Zorginstituut Nederland) [1] and age-specific hour earnings as published by Statistic Netherlands [6].

1. Hakkaart-van Roijen L, Van der Linden N, Bouwmans C, Kanters T, Swan Tan S. Manual for economic evaluations in health care [in Dutch]. Zorginstituut Nederland, 2016.

2. Kemmeren JM, Mangen MJJ, Van Duynhoven YTHP, Havelaar AH. Priority setting of foodborne pathogens. Bilthoven: 2006.

3. Care Institute Netherlands. Medine costs: price information: Zorginstituut Nederland; 2017. Available from: <https://www.medicijnkosten.nl/servicepagina/engelse-informatie/objectives>.

4. van den Brandhof WE, De Wit GA, de Wit MA, van Duynhoven YT. Costs of gastroenteritis in The Netherlands. Epidemiology and infection. 2004;132(2):211-21. Epub 2004/04/06. PubMed PMID: 15061495; PubMed Central PMCID: PMCPMC2870096.

5. Central Bureau for Statistics. Hourly wages of employees by profession, 2016 [in Dutch] 2016. Available from: <https://www.cbs.nl/nl-nl/maatwerk/2017/48/uurlonen-van-werknemers-naar-beroepsgroep-2016>.

6. Central Bureau for Statistics. Employment; jobs, wages, working hours, SBI2008; key figures 2018 [23 November 2018]. Available from: <http://statline.cbs.nl/Statweb/publication/?DM=SLNL&PA=81431NED&D1=2&D2>= 0-15&D3=0&D4=6-7&HDR=G3,G1,T&STB=G2&VW=T.
