# Supplementary material for: Incidence and economic burden of community-acquired gastroenteritis in the Netherlands: Does having children in the household make a difference?
Source: PLoS One. 2019 May 23;14(5):e0217347. doi: 10.1371/journal.pone.0217347 (PMC6532970; doi:10.1371/journal.pone.0217347)
Supplement: S2 Table — (DOCX) [file pone.0217347.s004.docx]

**S2 Table**. Population characteristics of the ESBLAT study population (before and after weighting) and the Dutch population.

|  | The Netherlands^a^  n=17,203,230 | |  | Study population  n=9,512 | | |  | Study population standardized^c^  N=9,512 | | |
| --- | --- | --- | --- | --- | --- | --- | --- | --- | --- | --- |
|  | N | % |  | N | % | Difference^b^ |  | N | % | Difference^c^ |
| Age (years) |  |  |  |  |  |  |  |  |  |  |
| 0-4 | 872,500 | 5.1 |  | 536 | 5.6 | +0.5 |  | 486 | 5.1 | 0.0 |
| 5-12 | 1,503,600 | 8.7 |  | 865 | 9.1 | +0.4 |  | 858 | 9.0 | +0.3 |
| 13-19 | 1,442,000 | 8.4 |  | 791 | 8.3 | -0.1 |  | 783 | 8.2 | -0.2 |
| 20-39 | 4,207,750 | 24.5 |  | 1,688 | 17.8 | -6.7 |  | 2,344 | 24.6 | +0.1 |
| 40-64 | 5,899,220 | 34.3 |  | 3,698 | 38.9 | +4.6 |  | 3,286 | 34.6 | +0.3 |
| 65-79 | 2,396,070 | 13.9 |  | 1,658 | 17.4 | +3.5 |  | 1,335 | 14.0 | +0.1 |
| 80+ | 882,090 | 5.1 |  | 276 | 2.9 | -2.2 |  | 420 | 4.4 | -0.7 |
| Gender (female) | 8,673,300 | 50.4 |  | 5,004 | 52.6 | +2.2 |  | 4,786 | 50.3 | -0.1 |
| Location of residence^d^ |  |  |  |  |  |  |  |  |  |  |
| Urban | 3,893,450 | 22.6 |  | 588 | 6.2 | -16.4 |  | 2,152 | 22.6 | 0.0 |
| Intermediate | 10,513,460 | 61.1 |  | 5,914 | 62.2 | +1.1 |  | 5,814 | 61.1 | 0.0 |
| Rural | 2,796,100 | 16.3 |  | 3,010 | 31.6 | +15.3 |  | 1,547 | 16.3 | 0.0 |

^a^Population on 1 January 2016

^b^Difference between the study population and the population in the Netherlands

^c^Standardized by age (five-year age groups), gender and urbanization (urban, intermediate, rural)

^d^Urban: ≥2500 addresses/km^2^, intermediate: 500-2500 addresses/km^2^, rural: <500 addresses/km^2^
